# Discussions of Cannabis Over Patient Portal Secure Messaging: Content Analysis

**DOI:** 10.2196/63311

**Published:** 2024-12-12

**Authors:** Vishal A Shetty, Christina M Gregor, Lorraine D Tusing, Apoorva M Pradhan, Katrina M Romagnoli, Brian J Piper, Eric A Wright

**Affiliations:** 1 Center for Pharmacy Innovation and Outcomes Geisinger Danville, PA United States; 2 Department of Health Promotion and Policy University of Massachusetts Amherst, MA United States; 3 Department of Population Health Sciences Geisinger Danville, PA United States; 4 Department of Medical Education Geisinger Commonwealth School of Medicine Scranton, PA United States; 5 Department of Bioethics and Decision Sciences Geisinger College of Health Sciences Gesinger Danville, PA United States

**Keywords:** patient portal, secure message, marijuana, patient-provider communication, message content, content analysis, United States, pain, anxiety, depression, insomnia, electronic messaging, electronic health record, EHR, cannabis

## Abstract

**Background:**

Patient portal secure messaging allows patients to describe health-related behaviors in ways that may not be sufficiently captured in standard electronic health record (EHR) documentation, but little is known about how cannabis is discussed on this platform.

**Objective:**

This study aimed to identify patient and provider secure messages that discussed cannabis and contextualize these discussions over periods before and after its legalization for medical purposes in Pennsylvania.

**Methods:**

We examined 382,982 secure messages sent by 15,340 patients and 6101 providers from an integrated health delivery system in Pennsylvania, United States, from January 2012 to June 2022. We used an unsupervised natural language processing approach to construct a lexicon that identified messages explicitly discussing cannabis. We then conducted a qualitative content analysis on a random sample of identified messages to understand the medical reasons behind patients’ use, the primary purposes of the cannabis-related discussions, and changes in these purposes over time.

**Results:**

We identified 1782 messages sent by 1098 patients (7.2% of total patients in the study) and 800 messages sent by 430 providers (7% of total providers in the study) as explicitly discussing cannabis. The most common medical reasons for use stated by patients in 190 sampled messages included pain or a pain-related condition (50.5% of messages), anxiety (13.7% of messages), and sleep (11.1% of messages). We coded 56 different purposes behind the mentions of cannabis in patient messages and 33 purposes in 100 sampled provider messages. In years before the legalization (2012-2016), patient and provider messages (n=20 for both) were primarily driven by discussions about cannabis screening results (38.9% and 76.5% of messages, respectively). In the years following legalization (2017-2022), patient messages (n=170) primarily involved seeking assistance to facilitate medical use (35.2% of messages) and reporting current use (25.3% of messages). Provider messages (n=80) were driven by giving assistance with medical marijuana access (27.5% of messages) and stating that they were unable to refer, prescribe or recommend medical marijuana (26.3% of messages).

**Conclusions:**

Patients showed a willingness to discuss cannabis use over patient portal secure messages and expressed interest in use after the legalization of medical marijuana. Some providers responded to patient inquiries with assistance in obtaining access to medical marijuana, while others cautioned patients on the risks of use. Insight into cannabis-related discussions through secure messages can help health systems determine opportunities to improve care processes around patients’ cannabis use, and providers should be supported to communicate accurate and consistent information.

## Introduction

Medical marijuana (defined as derivatives of the *Cannabis sativa* plant used to ease symptoms for medical conditions certified by a clinician and acquired through state-sanctioned dispensaries) [1] has been legalized in 38 US states, of which 24 have legalized recreational use as of 2024 [2]. In Pennsylvania, the Medical Marijuana Act was signed into law in 2016, legalizing the use of medical marijuana for 17 medical conditions [3]. There are now 24 “qualifying conditions” for which patients can claim eligibility to participate in the state’s medical marijuana program [4]. Across the United States, many patients are using marijuana to manage medical conditions, including intractable pain, anxiety and depression, and insomnia [5-10]. This patient use comes despite known risks associated with use, such as cannabis use disorder, impaired cognitive functioning and motor coordination, mood disorders, and exacerbation of psychotic disorders [11-15]. Patient use is also seen despite limited evidence for the efficacy of medical marijuana in treating many health conditions for which patients commonly use [16-18]. Given the potential consequences of cannabis (defined as any of several psychoactive preparations derived from the plant genus *Cannabis* used for recreational or medical purposes, which may or may not be legal depending on the state, preparation, and source) [19], misuse among patient populations, several recent studies have focused on documentation of cannabis use in electronic health records (EHRs) to identify patients who use and understand the nature of their use [20-22]. However, such effort has not been extended to other sources of unstructured textual clinical information, such as secure electronic messaging within patient portal systems.

Patient portal systems have been widely implemented across health care organizations in the United States [23]. Among organizations that have a patient portal, patient adoption is estimated to increase between 5% and 10% per year [24]. In addition, large multispecialty health systems have seen substantial increases in patient portal message volume within the last decade [25]. From 2020 to 2023, the number of patients with an active patient portal account at Geisinger, an integrated health delivery system in Pennsylvania, increased 59.3% from 533,660 to 850,208, and the number of patients who sent secure messages increased 146.3% from 223,630 to 550,740. These patients sent 1,178,288 and 1,832,288 total secure messages over this period, respectively. Secure messaging within patient portals enables asynchronous communication between patients and providers, which allows patients to express their thoughts and explain health-related behaviors in ways that cannot be sufficiently captured in standard EHR documentation [26-28]. Investigation of cannabis-related discussions by secure messaging can give health systems insight into the dynamics of patient use and reveal how providers navigate this topic with patients.

In this study, we aimed to identify patient and provider secure messages that discussed cannabis and contextualize these discussions around the legalization of medical marijuana in Pennsylvania. We used an unsupervised natural language processing approach to construct a lexicon to flag messages explicitly discussing cannabis. We then conducted a qualitative content analysis to understand the medical reasons behind patients’ use (if any) and the overall purposes of the cannabis-related discussions.

## Methods

### Study Population and Dataset

This study was conducted at Geisinger, one of the larger integrated health systems in the United States, serving approximately 1 million patients annually across 45 counties in central and northeast Pennsylvania. Geisinger comprises 9 acute care hospitals, an alcohol and chemical dependency treatment center, 133 specialty and primary care centers, 2 research centers, and an insurance company, Geisinger Health Plan (GHP) [29]. The study population included patients who had been flagged as cannabis users (medical or recreational) with at least one inpatient or outpatient encounter from January 2013 to June 2022, and at least one secure message sent a year before or a year after the date of their cannabis use indication. Messages were eligible for the analysis if they were designated in the “Patient Medical Advice Request” category of platform messages. These are patient-initiated messages which represent the primary dialogue between patients and providers. Flagged cannabis users were identified by discrete indications in the “Smart Data Element,” “Problem List,” “Encounter Diagnosis,” “Labs,” “Medications,” and “Social History” areas of the EHR (more details in Multimedia Appendix 1 for details on discrete indications of cannabis use). A total of 15,340 adult (age≥18 years) patients were included in the study population.

### Message Selection

Among the study population, all patient messages were sent a year before and a year after their discrete cannabis use identification, and all corresponding provider response messages were extracted (up to and including June 2022). To identify secure messages that discussed cannabis, we filtered messages that contained at least one mention of a cannabis-related keyword based on a constructed lexicon. We started with an initial lexicon derived from previous literature [22]. We excluded terms related to cannabinoid-based prescription drugs such as “Epidiolex,” “Marinol,” and “Dronabinol,” as the focus of our analysis was on plant-based cannabis products. To refine our lexicon and mitigate against the misclassification of messages, we constructed word similarity lists using Word2Vec. Word2vec is an unsupervised natural language processing method that generates vector representations for each word in a set of documents (eg, secure messages) [30]. Words that appear in similar contexts will have similar representations. For each word in our initial lexicon, we generated the top 10 words that most frequently appear in similar contexts (more details in Multimedia Appendix 2 for the initial lexicon and generated similarity lists).

Members of the lexicon were removed if no similarity list was generated (ie, the word appeared fewer than 5 times in all messages) or if its similarity list did not contain at least two words related to cannabis. A word in a similarity list was added to the lexicon if it had a meaning related to cannabis or was a misspelling of a member of the lexicon. Note the similarity lists for the term “pot” revealed that it appeared frequently as cannabis-related words but also in unrelated contexts. To minimize misclassification in these cases, we removed all messages that contained “neti pot,” “netti pot,” “netty pot,” and “netting pot.” Our final lexicon included the following words: “cannabis,” “cannabinoid,” “cbd,” “edible,” “edibles,” “hemp,” “ilera,” “indica,” “mariajuana,” “marihuana,” “marijuana,” “marijuanna,” “marj,” “marjuana” “mj,” “mmj,” “pot,” “sativa,” “thc,” “tincture,” “tinctures,” “weed.” Text processing was conducted using Python (version 3.8.10; Python Software Foundation), and Word2vec representations were generated using the library Gensim [31].

### Codebook Development

Messages discussing cannabis were organized into four groups: (1) patient messages sent from 2012-2016, (2) patient messages sent from 2017 to 2022, (3) provider messages sent from 2012 to 2016, and (4) provider messages sent from 2017 to 2022. Messages were organized by the years in which they were sent in order to separate those that occurred before and after the state medical marijuana program was signed into law in Pennsylvania (2017 being the first full year after the passing of the law) [3]. One member of the research team (VAS) reviewed a random sample of 10 messages from each group to identify broad concepts in the text related to patient cannabis use and the purpose behind the mention of cannabis. This step was repeated until a point of saturation in concepts was reached [32]. 50 patient messages and 50 provider messages were reviewed (20 messages from 2012-2016 groups, 30 from 2017-2022 groups). This process produced 2 initial codebooks for patient and provider messages separately. Each codebook was reviewed by members of the research team.

Our analysis of message content was conducted using a random sample of at least 10% of messages from each group (minimum of 20 messages) to reach sufficiency for analysis [33]. Using the initial codebooks, two coders (VAS and CMG) independently coded 5 messages from each group. Discrepancies in codes were discussed, and modifications were made to the codebook. This process was repeated until an interrater reliability (Cohen κ) [34] of at least 0.75 was achieved; two rounds of double coding were needed to achieve sufficient interrater reliability [35]. A κ of 0.66 was achieved in the first round, and 0.81 was achieved in the second round. The remaining messages in each of the four message groups were then split evenly between the two coders for single coding. After each coder had reviewed their respective half of messages for a group, suggestions for new codes and modifications to existing codes were discussed between the coders and a third member (LDT). Changes were made to the appropriate codebook, and messages in that group were recoded over iterative rounds until no changes were suggested. This process was repeated for each message group to finalize codebooks.

For patient messages, five primary content categories were coded: (1) correctly classified message (ie, the keyword which flagged the message as pertaining to a plant-based product), (2) message author (patient or nonpatient), (3) time of cannabis use, (4) reason for cannabis use, and (5) purpose of cannabis mention in message. “Time of cannabis” use codes were mutually exclusive. An exception was made if a message described the use of multiple different cannabis products. Multiple “reasons for cannabis use” and “purposes of the cannabis mention” could be coded in each message. “Reason for cannabis use” codes included explicitly named health issues patients used, were using, or wanted to use cannabis for. “Purpose of the cannabis mention” codes were determined based on the coders’ judgment of the primary drivers behind the patient bringing up cannabis in the message.

For provider messages, four primary content categories were coded: (1) correctly classified message, (2) message recipient (patient or nonpatient), (3) reason for cannabis use, and (4) purpose of cannabis mention. “Reason for cannabis use” codes were determined based on the provider’s description of what the patient had used or wanted to use for (more details in Multimedia Appendix 3 for the finalized codebooks with code definitions).

### Analysis and Synthesis

Frequencies were calculated for all finalized codes. We used two proportion *z* tests to calculate differences in code frequencies between messages sent from 2012-2016 and messages sent from 2017-2022. We conducted a thematic analysis based on frequency patterns in codes to synthesize the information collected from messages [33]. Each coder independently rereviewed example messages of coded categories and analyzed code frequencies to construct qualitative memos in which themes were chosen to capture how patients and providers have been using secure messages to discuss cannabis, with a particular focus on how message content reflected the legal status of medical marijuana. Each coder justified chosen themes by citing the data that supported a theme and by describing the broader implications of the theme in terms of patient-provider interactions around cannabis use. Coders comprehensively discussed themes to come to a consensus, and themes were reviewed and further refined with the full research team.

### Ethical Considerations

This study was approved by the Geisinger Institutional Review Board (IRB00008345). All procedures were performed in accordance with the relevant guidelines and regulations.

## Results

### Message, Patient, Provider Characteristics

**Figure 1:**
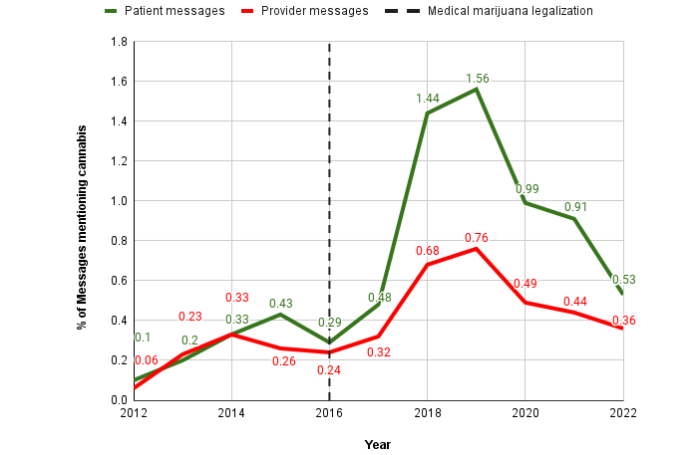
Proportion of all secure messages that include a mention of cannabis from 2012-2022.

Among 206,699 messages sent by 15,340 patients from 2012-2022, we identified 1782 messages (0.9%) from 1098 patients (7.2%; 1.6 average messages per patient) as explicitly mentioning cannabis (either medical marijuana or recreational). Among 176,283 messages sent by 6101 providers from 2012-2022, we identified 800 messages (0.5%) from 430 providers (7.0%; 1.9 average messages per provider) as explicitly mentioning cannabis. The proportion of patient messages mentioning cannabis increased from 0.3% from 2012-2016 to 0.97% from 2017-2022 (Figure 1). The proportion of provider messages mentioning cannabis increased from 0.25% from 2012-2016 to 0.51% from 2017-2022. The largest increase in the volume of patient and provider messages mentioning cannabis occurred from 2017 to 2018 (57 to 251, 340.4% and 34 to 108, 217.6% increase, respectively). This coincided with the opening of the first medical marijuana dispensary in Pennsylvania in February 2018 [36]. For all patient and provider messages from 2017 to 2018, volume increased by 47.5% (11,835 to 17,454) and 48.3% (10,638 to 15,773), respectively (more details in Multimedia Appendices 4 and 5 for trends in patient and provider message volume over time).

Patients in the total study population were primarily White non-Hispanic (84.9%, 13,028/15,340), female (60.6%, 9301/15,340), had a mean age of 39.7 (SD 16), and were insured under GHP (37.9%, 5812/15,340). Patient messages were primarily sent by those insured under GHP (40.4%, 83,644/206,699), a commercial plan (24.2%, 50,081/206,699), Medicare (16.9%, 35,029/206,699), and Medicaid (12.7%, 26,323/206,699). Among patients who either sent or only received a message mentioning cannabis (n=1098 and n=150, respectively), most were White non-Hispanic (90%, 1118/1248), female (63%, 786/1248), had a mean age of 41.8 (SD 15.6), and were insured under GHP (38.9%, 486/1248). Patient messages discussing cannabis were primarily sent by those insured under GHP (38%, 678/1782), a commercial plan (26.5%, 472/1782), Medicare (19.3%, 344/1782), and Medicaid (10.4%, 185/1782). Provider messages to patients in the study population were primarily sent from family medicine (38.9%, 68,511/176,283), obstetrics and gynecology (6.8%, 12,056/176,283), gastroenterology (5.1%, 9078/176,283), neurology (5.1%, 8963/176,283), and internal medicine (4.7%, 8350/176,283). Messages were primarily sent by physicians (33.7%, 59,488/176,283), registered nurses (15.3%, 26,943/176,283), and physician assistants (13.5%, 23,865/176,283). Provider messages discussing cannabis were primarily sent from family medicine (50.9%, 407/800), neurology (13.1%, 105/800), internal medicine (5.1%, 41/800), gastroenterology (4.1%, 33/800), and psychiatry (3.4%, 27/800). Messages were primarily sent by physicians (61.1%, 489/800), physician assistants (15.5%, 124/800), and registered nurses (11%, 88/800).

### Message Content Frequencies

In the coded patient messages sent from both 2012-2016 and 2017-2022, at least half included a health-related reason for having used, using, or wanting to use cannabis (Table 1). Patients named 34 different reasons for use, though there may be overlap between some, such as anxiety (1/18, 5.6% of messages in 2012-2016; 25/162, 15.4% in 2017-2022) and racing thoughts (1/162, 0.6% of messages in 2017-2022). Note that Table 1 only includes the three most prevalent categories.

**Table 1:** Frequency of patient message content categories related to cannabis by year of message.

Content category		2012-2016 (N=20), n (%)	2017-2022 (N=170), n (%)	*P* value^a^
**Correctly classified message**				
	Yes	18 (90)	162 (95.3)	—^b^
	No, not related to cannabis	1 (5)	8 (4.7)	—
	No, reference to Rx	1 (5)	—	—
**Message author^c^**				
	Patient	17 (94.4)	153 (94.4)	—
	Patient proxy	1 (5.6)	9 (5.6)	—
**Time of use^c,d^**				
	Past, but not current	5 (27.8)	18 (11.1)	.04
	Current	5 (27.8)	53 (32.7)	.67
	Interest or intent to use	5 (27.8)	80 (49.4)	.08
	None	3 (16.7)	18 (11.1)	.48
**Reason for use^c,e^**				
	Not specified	9 (50)	70 (43.2)	.58
	Pain or pain condition	12 (66.7)	84 (51.9)	.23
	Anxiety	1 (5.6)	25 (15.4)	.26
	Sleep	—	21 (13)	.10
**Purpose of cannabis mention^c,e^**				
	Seeking assistance to facilitate use	4 (22.2)	57 (35.2)	.27
	Seeking guidance on recommendation to use	1 (5.6)	23 (14.2)	.31
	Statement of plan to use	—	17 (10.5)	.15
	Reporting current use	3 (16.7)	41 (25.3)	.42
	Reporting no current use	5 (27.8)	13 (8)	.01
	Discussion of cannabis screening results	7 (38.9)	9 (5.6)	<.001

^a^*P* values were calculated using two proportion *z* tests.

^b^Not available.

^c^Percentages are based on values of correctly classified messages (ie, n=18 for 2012-2016 messages, n=162 for 2017-2022 messages).

^d^Some patients indicated using different cannabis products at varying times, in which case they were assigned to multiple “Time of use” categories.

^e^Only includes a subset of coded categories.

Reason for use of coded inpatient messages (more details in Multimedia Appendix 6 for frequencies of all coded categories). The most frequently mentioned reason for use in both 2012-2016 and 2017-2022 was pain or a pain-related condition, which included general pain (7/18, 38.9% of messages in 2012-2016; 49/162, 30.2% in 2017-2022), pain in a specific body part (2/18, 11.2% of messages in 2012-2016; 21/162, 13.1% in 2017-2022), migraine (3/18, 16.7% of messages in 2012-2016; 6/162, 3.7% in 2017-2022), and fibromyalgia (8/162, 4.9% of messages in 2017-2022). We coded 56 different purposes behind the mentions of cannabis in patient messages. From 2017-2022, messages about cannabis were largely driven by patients seeking assistance to facilitate medical marijuana use (57/162, 35.2%), which included patients requesting formal approval to use (ie, physical documentation or a diagnosis of a medical marijuana qualifying condition to be reflected in their EHR; 32/162, 19.8%), patients asking for help with access, such as a request to help with accessing a medical marijuana card (9/162, 5.6%), patients asking for a referral to a certifying provider (13/162, 8%), and patients asking for a medical marijuana prescription (6/162, 3.7%). Note that “prescription” is the language used by patients, but providers cannot prescribe medical marijuana (for a description of Pennsylvania’s medical marijuana access process for patients; more details in Multimedia Appendix 7). From 2012-2016, a significantly higher proportion of messages were about cannabis screening results (7/18, 38.9%) and involved patients reporting no current cannabis use (5/18, 27.8%) compared with messages from 2017-2022 (9/162, 5.6% and 13/162, 8%; *P*<.01 and *P*=.01).

Fewer than 20% of provider messages from both 2012-2016 and 2017-2022 specified a reason for a patient’s use of cannabis (Table 2). Providers indicated 10 different reasons for patient use, 7 of which were named in patient messages. Like patient messages, pain or a pain-related condition was the most frequently mentioned reason for use (2/17, 11.8% of messages in 2012-2016; 8/80, 10% in 2017-2022), with general pain being the most common of these mentions (1/17, 5.9% of messages in 2012-2016; 7/80, 8.8% in 2017-2022). We coded 33 different purposes behind the mentions of cannabis in provider messages. The most common purpose from 2017-2022 was to assist patients in accessing medical marijuana (22/80, 27.5%). Providers also frequently stated they were unable to refer, prescribe, or recommend medical marijuana (21/80, 26.3%), citing health system policy and the limitations in the scope of their practice as primary justifications (8/80, 10%, and 7/80, 8.8%, respectively). From 2012-2016, a significantly higher proportion of messages from providers involved information about a cannabis screening result (13/17, 76.5%) compared with messages from 2017-2022 (7/80, 8.8%; *P<*.01). These messages from 2012-2016 included the provider informing the patient of a positive result or that they had violated a medication use agreement to not use cannabis (11/17, 64.7% and 7/17, 41.2%, respectively).

**Table 2:** Frequency of provider message content categories related to cannabis by year of message.

Content category		2012-2016 (N=20), n (%)	2017-2022 (N=80), n (%)	*P* value^a^
**Correctly classified message**				
	Yes	17 (85)	80 (100)	—^b^
	No, not related to cannabis	3 (15)	—	—
	No, reference to Rx	—	—	—
**Message recipient^c^**				
	Patient	17 (100)	75 (93.8)	—
	Patient proxy	—	5 (6.3)	—
**Reason for use^c,d^**				
	Not specified	14 (82.4)	65 (81.3)	.91
	Pain or pain condition	2 (11.8)	8 (10)	.83
	Anxiety	—	4 (5)	.35
	Sleep	—	1 (1.3)	.65
**Purpose of cannabis mention^c,d^**				
	Providing assistance with medical marijuana access	—	22 (27.5)	.01
	Explanation of potential drug-to-drug interaction	2 (11.8)	12 (15)	.73
	Explanation of other associated adverse events	—	8 (10)	.17
	Unable to refer, prescribe, or recommend use	2 (11.8)	21 (26.3)	.20
	Providing information on cannabis screening	13 (76.5)	7 (8.8)	<.001

^a^*P* values were calculated using two proportion *z* tests.

^b^Not available.

^c^Percentages are based on values of correctly classified messages (ie, n=18 for 2012-2016 messages, n=162 for 2017-2022 messages).

^d^Only includes a subset of coded categories.

### Thematic Analysis

Our analysis of the frequency patterns in message content produced 6 themes. The themes reflect the primary takeaways of the coded data, with differences between message groups related to the legalization of medical marijuana being a key focus. Not all coded categories were used as a part of this analysis. Representative patient and provider messages are given for each theme. Note that select quotes are given for each theme, and those that contain inaccurate information were labeled with [sic].

1. Many patient reasons for using cannabis were expressed in messages, with mixed connections to Pennsylvania qualifying conditions. Between patient and provider messages, 37 distinct reasons for patients having used or wanting to use medical marijuana were named. This includes multiple variations of the same type of health issue. For example, 11 pain-related issues were named. Out of the 37 reasons, 19 can be linked to a Pennsylvania qualifying condition for medical marijuana, and only 9 of 27 reasons if pain-related issues are considered together (more details in Multimedia Appendix 8 for a list of medical marijuana qualifying conditions in Pennsylvania). While patients primarily mentioned reasons for medical marijuana use that could be considered qualifying conditions, such as pain and anxiety, there were a number of cases where patients discussed use for purposes that had no obvious connection to a qualifying condition. For example, sleep was a commonly cited reason for a patient’s use or interest in use:

I wanted to talk about the possibility of getting a marijuana card. I’ve been having a hard time falling asleep and staying asleep. I’ve tried trazodone, but I don’t like the “hung over” feeling the next day. How do I go about getting this? Thanks.

Providers did not often indicate a patient’s reason for use, and most of these few instances referenced pain or anxiety. When these reasons were mentioned, providers tended to endorse the use or relay the positive experiences of other patients. One provider wrote, “Medical marijuana can certainly be helpful to reduce chronic pain…” Another wrote, “Unfortunately I do not have expertise in marijuana. We have heard a lot of success with it for pain, anxiety control, etc”

2. After the legalization of medical cannabis, patients expressing interest in use was the primary driver of cannabis-related discussions. Nearly 50% of patient messages after 2016 expressed interest in using or explicit intent to use medical marijuana. Patients primarily expressed their interest in using medical marijuana through direct requests to a provider for approval to use:

The doctor I contacted regarding medical marijuana needs a diagnosis code for my anxiety and recommendation from a doctor before I can schedule an appointment with him. Is that something you can provide me?

Patients also expressed their interest by seeking recommendations from a provider on whether to use:

... I have heard a lot lately about medical marijuana for various conditions and was wondering what your opinion on it is… I guess I’m wondering if it could be something that would be able to help my mental health issues, as well as my physical ones…

Other patients were more forthcoming, explicitly stating their plans to use medical marijuana:

Just to inform you, I got my medical marijuana card. They are starting with a CBD tincture. I will be going to pick it up today or tomorrow.

3. In response to patient interest in using medical marijuana, some providers tried to support this demand while others took a more cautious approach. In the face of receiving patient inquiries about accessing medical marijuana and requests for approval to use, many providers attempted to give some level of assistance. This assistance ranged from simply directing a patient to the state medical marijuana program website to giving a list of certifying providers to outright certifying patients in limited cases. Providers who assisted in this way were primarily from the family medicine department. A physician assistant from family medicine sent the following message:

Referrals are not needed to obtain a medical marijuana card - insurance will not pay for this type of treatment. You just need to schedule an intake with a doctor who has gone through the credentialing to evaluate you for this. If you are deemed an appropriate candidate, you then go to a pharmacy [sic] where the medical marijuana is dispensed. [Name of physician] is a local doctor who can do this. I copied and pasted his information below.

At the same time, other providers took a more cautious approach in guiding patients around medical marijuana use. This included informing about potential drug interactions or other adverse outcomes and recommendations to not use medical marijuana. The distribution of departments that these messages were sent from was heterogeneous, including family medicine, internal medicine, psychiatry, psychology, rheumatology, neurology, cardiology, pharmacy, gastroenterology, and obstetrics and gynecology. For instance, a physician from the neurology department gave a warning to a patient:

In general, I do not recommend the use of medical marijuana for people with cognitive concerns given that it can have some brain impairing effects. CBD alone may or may not have brain impairing side effects and until that is clarified, I also don’t in general recommend its use in those with cognitive concerns.

4. Patients expressed a willingness to admit and discuss use after 2016, a contrast from previous years characterized by patients distancing themselves from use. From 2012-2016, more messages included statements about current cannabis nonuse than current use. Patients tended to be defensive and denied any cannabis use in response to a positive screening result:

I think you misunderstood! I was not lying, since I signed the agreement I stopped the pot because this pain is real and pot is not worth the pain, which is why I said it’s an old habit and that it will not be in my urine anymore…

The tenor and content of provider messages from 2012 to 2016 may help to explain these patient responses. Cannabis was primarily brought up by providers to inform patients of a positive screening result and that they had violated their medication use agreement. In 2013, a family medicine physician sent the following message:

Marijuana ingestion is not accidental and is in violation of your medication usage agreement. Simply put, this clinic will never be writing for any controlled substance ever again.

However, more messages included statements about current use than current nonuse from 2017 to 2022. Patients were transparent about cannabis use, bringing it up on their own to provide more context around their situation. For instance, one patient was upfront about their use and why they use it: “I smoke marijuana due to my anxiety, and it helps me sleep…”

5. In the early years of legalized medical marijuana, some providers signaled that they were not in a position or were unwilling to engage in discussions about medical marijuana. Over 25% of provider messages from 2017-2022 included a statement that the provider was unable to refer, prescribe, or recommend medical marijuana use. Despite this lack of engagement from providers on the topic of medical marijuana, 52.5% (42/80) of these messages still attempted to support patients’ requests. In the following message, a provider cites their own limited knowledge and the absence of a health system medical marijuana policy to abstain from making a clear recommendation. It is important to note that Geisinger has had a medical marijuana policy since 2017 acknowledging the status of marijuana as a Schedule I substance from the federal Controlled Substance Act and, in accordance, does not advocate for medical marijuana use. However, it does allow for self-administration of legal products under medical supervision within facilities and allows providers to share information about medical marijuana and register with the Commonwealth to certify patients with medical conditions recognized by Pennsylvania. While this provider appears to be unaware of the policy, they still try to direct the patient toward the relevant resources:

I’m not sure if marijuana would help you with your symptoms, and I personally do not have any experience with medical marijuana. There is no medical marijuana policy in Geisinger [sic], but there is a government program (Pennsylvania’s medical marijuana program). You can go to their website and find out about the locations and providers who prescribe [sic] medical marijuana… [URL]

## Discussion

### Principal Findings

We identified 1782 messages from 1098 patients and 800 messages from 430 providers using a Word2Vec-constructed lexicon of cannabis key terms as explicitly discussing cannabis (both recreational and medical marijuana). A further content and thematic analysis revealed changes in the purposes behind discussions of cannabis between years before the legalization of medical marijuana in Pennsylvania and years after legalization. Before legalization, patients and providers primarily discussed cannabis in the context of a patient’s positive toxicology screening result. In the years after legalization, patients sought access to medical marijuana and guidance around its use, while providers were split between supporting requests and cautioning patients on the risks of cannabis (ie, informing about potential drug interactions or other adverse outcomes and recommending not to use medical marijuana). Patients were also willing to disclose that they were currently using cannabis, often specifying the health-related issue they were using it for.

We found in our content analysis that just under half of patient messages from 2017 to 2022 expressed interest in using cannabis for a health-related reason, indicating that some patients are using the patient portal as a vehicle to engage providers about cannabis as a clinical option. While cannabis-related discussions over secure messaging have not been explored, previous studies have examined the general message content of other patient populations and topics, such as COVID-19 [27], veterans [28], and veterans with diabetes [26]. These studies found patients most frequently sent messages that were transactional (eg, appointment scheduling, administrative requests), followed by informational (eg, updates on health status or care) or interactional (eg, request for input on health-related issues) [26,28]. This is consistent with our own findings on the content of patient messages after 2016 if seeking assistance to facilitate medical marijuana use is considered transactional (eg, request for an appointment with or a referral to a certifying provider, request for a diagnosis code that reflects a qualifying condition), reporting current cannabis use or nonuse is considered informational, and seeking guidance on cannabis use is considered interactional. Our observations of patients’ use of secure messaging suggest after legalization, some patients viewed medical marijuana similarly to more clinically established medications and treatment options. As a result, these patients turned to providers as a primary point of access as they would for other therapies. Several studies have reported sharp increases in cannabis use across various populations in the immediate aftermath of state legalization (medical or recreational) [37-39]. In Pennsylvania and other states that have recently legalized medical marijuana, it should be expected patients will express their interest in the use, and secure messaging may be used to express this interest.

In several states with medical marijuana programs, providers have expressed concern over the risks of patient use, particularly with respect to drug interactions [40-43]. This parallels our own finding of providers cautioning patients around use, including by informing them about potential drug interactions. Much of the concern from providers may be the result of uncertainty and an overall lack of knowledge on medical marijuana [40,41,43,44]. Despite this uncertainty, some providers believe marijuana has medical efficacy for certain conditions, such as intractable pain and terminal illness [41,43]. This may explain our finding that in over half of the messages where a provider claimed they could not refer, prescribe [sic]*,* or recommend medical marijuana, they followed up with assistance for the patient to access medical marijuana. Patients in our study primarily named pain, anxiety, and sleep as reasons for using or wanting to use medical marijuana, and these are the most commonly reported reasons for use in patients across the United States [10,45]. Providers may be more comfortable supporting use for reasons about which they are frequently approached [43,46]. However, patients in our study brought up numerous other reasons for use that would not directly be considered a qualifying condition in Pennsylvania. Some of these reasons included cramps, periods, inflammation, skin irritation, mood swings, vertigo, tremors, and attention deficit disorder. Provider caution is warranted in the absence of an informed position, particularly for conditions that do not qualify for use under a state’s medical marijuana program. However, some patients have reported frustration and distrust in providers who come across as overly negative or uneducated about medical marijuana [47,48]. Health systems should support providers with updated evidence-based guidelines on medical marijuana use for medical conditions [17], and providers must be prepared to communicate this information over mediums such as secure messaging to respond to patient inquiries with appropriate and accurate information.

Stigma exists as a part of the experience of medical marijuana users, which can lead to stress, anxiety, isolation, and avoidance of interacting with health care professionals [47,49-51]. Despite California legalizing medical marijuana in 1996, patient users have reported fears of being stigmatized more than a decade after legalization, including by their own physicians [50]. We found more patients openly admitted to using cannabis in postlegalization years versus prelegalization years. However, many patients may choose not to discuss their cannabis use over secure messaging due to a variety of factors, including stigma. It is unknown whether secure messaging as a communication platform facilitates or hinders patient disclosure of sensitive information such as cannabis use. A recent investigation found among patients with hypertension, diabetes, or both, younger patients were less likely to share clinical updates using secure messaging than older patients, while men were less likely to seek medical guidance than women [52]. At the same time, a study from California found that the population of medical marijuana users in the state is composed of a higher proportion of males and skewed younger than the general population [45]. While there may be many patients who are not disclosing their cannabis use [51], the patient portal platform may uniquely select patients who are more willing to disclose sensitive information. Patients with access to a patient portal account are more likely to have a primary care clinician, health insurance, and higher educational attainment than patients without access [53]. Lower socioeconomic status has been associated with higher levels of patient distrust of physicians [54], so patients who are less willing to share information may not be as represented in a population of patient portal users. Still, in messages sent before legalization, we found cannabis was primarily discussed in the context of positive screening results when a patient was “caught” rather than when the patient volunteered information on their use themselves. A previous investigation found evidence of patients in Pennsylvania withholding cannabis use information before legalization [55]. This shift in tenor prelegalization versus postlegalization may signal a change in patient attitudes toward sharing information related to cannabis use. Future studies should examine changes in cannabis use disclosure over time in states that recently legalized cannabis (for medical or recreational use), as well as the potential role secure messaging plays in facilitating disclosure.

### Limitations

Our study is the first to analyze the content of secure messages to understand cannabis-related discussions between patients and providers, but it is limited in several ways. First, of the messages we identified as explicitly discussing cannabis, relatively few occurred during the prelegalization period, likely because of the illegality of cannabis for any reason in the state during this period, as well as a lower overall message volume compared to recent years. The low message volume limited the concepts we were able to derive from our content analysis. However, among the messages we did analyze, a considerable proportion (7/18, 38.9% for patients and 13/17, 76.5% for providers) were related to discussions of cannabis screening results and the consequences of those results. This may be a common pattern in the message content for discussions of any illicit substance. Future work is needed to compare the volume and content of secure messages discussing cannabis versus other illicit substances, particularly as states continue to enact medical and recreational marijuana policies as well as after cannabis becomes a Schedule III substance. Second, our data includes messages sent a year before and a year after a patient in the study population had been flagged as a cannabis user in their EHR. It is possible that the patient message content we observed was influenced by the fact these patients had been flagged at some point as cannabis users. For example, patients who send cannabis-related messages after being flagged may be more open to discussing their use, and patients who express interest in cannabis over secure messages may be easier to flag when they eventually start using. Third, we focused our analysis at the message level, as our objective was to identify and contextualize explicit mentions of cannabis in individual messages. However, by excluding replies in our analysis, there may be messages in which we left out the full context behind the purpose of the cannabis mention. Excluding replies without an explicit mention of cannabis also means we are likely underestimating the number of “true” messages that discuss cannabis. However, we found that messages often included multiple topics and that replies may not address cannabis at all, even if the initial message explicitly mentioned cannabis. Future studies can expand on this work by analyzing entire message threads related to cannabis to understand the dynamics of patient-provider conversations on this topic, including how specific patient inquiries and disclosures correlate with specific provider responses. Finally, medical marijuana is currently legal in Pennsylvania, but the recreational use of cannabis is not as of this writing (December 2024). Further research is necessary to determine the generalizability of these findings to states with legal recreational marijuana or to different patient populations (ie, English as a second language patients or minors).

### Conclusions

We identified patient and provider secure messages at an integrated health delivery system discussing cannabis use and performed a content analysis to further contextualize these messages around the legalization of medical marijuana. After legalization in Pennsylvania, patients expressed interest in medical marijuana use and a willingness to disclose information related to their use. Providers were split in their responses to patient inquiries, with some aiding with access to medical marijuana while others emphasized its risks to patients. Secure messaging through the patient portal offers a channel through which providers can engage patients about cannabis use, but health systems can support providers in communicating consistent and appropriate guidance to prevent harmful use, particularly as patient portal adoption grows and providers experience a greater burden to respond to patient messages. Health systems should also continue to explore ways in which secure messaging can be used to facilitate patient sharing of cannabis use information to optimize their health care.

## Appendix

Multimedia Appendix 1 Algorithms for identifying discrete indications of cannabis use in electronic health records.

Multimedia Appendix 2 Word2vec word similarity lists (with cosine similarity scores) for cannabis lexicon.

Multimedia Appendix 3 Final patient and provider cannabis codebooks.

Multimedia Appendix 4 Volume of secure messages that include a mention of cannabis from 2012-2021.

Multimedia Appendix 5 Volume of messages among all patients in study population and responding providers from 2012-2021.

Multimedia Appendix 6 Frequencies for all patient and provider code categories.

Multimedia Appendix 7 Medical marijuana access process in Pennsylvania.

Multimedia Appendix 8 Pennsylvania medical marijuana qualifying conditions from 2016-2024.

## Abbreviations

EHR: electronic health record
GHP: Geisinger Health Plan

## References

[1] Mayo Clinic (2021). Medical marijuana.

[2] National Conference of State Legislatures State medical cannabis laws.

[3] (2016). Medical Marijuana Act - Enactment. Pennsylvania General Assembly.

[4] Pennsylvania Medical Marijuana Program Pennsylvania Department of Health.

[5] Kosiba JD, Maisto SA, Ditre JW (2019). Patient-reported use of medical cannabis for pain, anxiety, and depression symptoms: Systematic review and meta-analysis. Soc Sci Med.

[6] Jennings JM, Johnson RM, Brady AC, Dennis DA (2020). Patient perception regarding potential effectiveness of cannabis for pain management. J Arthroplasty.

[7] Salazar CA, Tomko RL, Akbar SA, Squeglia LM, McClure EA (2019). Medical cannabis use among adults in the Southeastern United States. Cannabis.

[8] Ryan-Ibarra S, Induni M, Ewing D (2015). Prevalence of medical marijuana use in California, 2012. Drug Alcohol Rev.

[9] Boehnke KF, Scott JR, Litinas E, Sisley S, Williams DA, Clauw DJ (2019). Pills to pot: observational analyses of cannabis substitution among medical cannabis users with chronic pain. J Pain.

[10] Azcarate PM, Zhang AJ, Keyhani S, Steigerwald S, Ishida JH, Cohen BE (2020). Medical reasons for marijuana use, forms of use, and patient perception of physician attitudes among the US population. J Gen Intern Med.

[11] Leung J, Chan GCK, Hides L, Hall WD (2020). What is the prevalence and risk of cannabis use disorders among people who use cannabis? a systematic review and meta-analysis. Addict Behav.

[12] Karila L, Roux P, Rolland B, Benyamina A, Reynaud M, Aubin H, Lançon Christophe (2014). Acute and long-term effects of cannabis use: a review. Curr Pharm Des.

[13] Volkow ND, Baler RD, Compton WM, Weiss SR (2014). Adverse health effects of marijuana use. N Engl J Med.

[14] Hall W (2015). What has research over the past two decades revealed about the adverse health effects of recreational cannabis use?. Addiction.

[15] Hill KP (2017). Cannabis use and risk for substance use disorders and mood or anxiety disorders. JAMA.

[16] Levinsohn EA, Hill KP (2020). Clinical uses of cannabis and cannabinoids in the United States. J Neurol Sci.

[17] Abrams DI (2018). The therapeutic effects of cannabis and cannabinoids: An update from the National Academies of Sciences, Engineering and Medicine report. Eur J Intern Med.

[18] Whiting PF, Wolff RF, Deshpande S, Di Nisio M, Duffy S, Hernandez AV, Keurentjes JC, Lang S, Misso K, Ryder S, Schmidlkofer S, Westwood M, Kleijnen J (2015). Cannabinoids for medical use: A systematic review and meta-analysis. JAMA.

[19] World Health Organization Cannabis. Alcohol, Drugs and Addictive Behaviours.

[20] Lapham GT, Matson TE, Carrell DS, Bobb JF, Luce C, Oliver MM, Ghitza UE, Hsu C, Browne KC, Binswanger IA, Campbell CI, Saxon AJ, Vandrey R, Schauer GL, Pacula RL, Horberg MA, Bailey SR, McClure EA, Bradley KA (2022). Comparison of medical cannabis use reported on a confidential survey vs documented in the electronic health record among primary care patients. JAMA Netw Open.

[21] Matson TE, Carrell DS, Bobb JF, Cronkite DJ, Oliver MM, Luce C, Ghitza UE, Hsu CW, Campbell CI, Browne KC, Binswanger IA, Saxon AJ, Bradley KA, Lapham GT (2021). Prevalence of medical cannabis use and associated health conditions documented in electronic health records among primary care patients in Washington state. JAMA Netw Open.

[22] Sajdeya R, Mardini MT, Tighe PJ, Ison RL, Bai C, Jugl S, Hanzhi G, Zandbiglari K, Adiba FI, Winterstein AG, Pearson TA, Cook RL, Rouhizadeh M (2023). Developing and validating a natural language processing algorithm to extract preoperative cannabis use status documentation from unstructured narrative clinical notes. J Am Med Inform Assoc.

[23] Heath S Patient Engagement HIT. Patient Portal Adoption Tops 90%, But Strong Patient Use is Needed.

[24] Schillinger D, McNamara D, Crossley S, Lyles C, Moffet HH, Sarkar U, Duran N, Allen J, Liu J, Oryn D, Ratanawongsa N, Karter AJ (2017). The next frontier in communication and the ECLIPPSE study: Bridging the linguistic divide in secure messaging. J Diabetes Res.

[25] North F, Luhman KE, Mallmann EA, Mallmann TJ, Tulledge-Scheitel SM, North EJ, Pecina JL (2020). A retrospective analysis of provider-to-patient secure messages: how much are they increasing, who is doing the work, and is the work happening after hours?. JMIR Med Inform.

[26] Robinson SA, Zocchi M, Purington C, Am L, DeLaughter K, Vimalananda VG, Netherton D, Ash AS, Hogan TP, Shimada SL (2023). Secure messaging for diabetes management: content analysis. JMIR Diabetes.

[27] Alpert JM, Campbell-Salome G, Gao C, Markham MJ, Murphy M, Harle CA, Paige SR, Krenz T, Bylund CL (2022). Secure messaging and COVID-19: a content analysis of patient-clinician communication during the pandemic. Telemed J E Health.

[28] Shimada SL, Petrakis BA, Rothendler JA, Zirkle M, Zhao S, Feng H, Fix GM, Ozkaynak M, Martin T, Johnson SA, Tulu B, Gordon HS, Simon SR, Woods SS (2017). An analysis of patient-provider secure messaging at two Veterans Health Administration medical centers: message content and resolution through secure messaging. J Am Med Inform Assoc.

[29] Geisinger About Geisinger.

[30] Mikolov T, Chen K, Corrado G, Dean J Efficient estimation of word representations in vector space.

[31] Rehurek R, Sojka P (2010). Software framework for topic modelling with large corpora. https://www.researchgate.net/publication/255820377_Software_Framework_for_Topic_Modelling_with_Large_Corpora.

[32] Taylor SJ, Bogdan R, DeVault ML (2015). Introduction to Qualitative Research Methods: A Guidebook and Resource.

[33] Pope C, Ziebland S, Mays N, Pope C, Mays N (2006). Analysing Qualitative Data.

[34] Cohen J (1960). A coefficient of agreement for nominal scales. Educational and Psychological Measurement.

[35] Viera AJ, Garrett JM (2005). Understanding interobserver agreement: the kappa statistic. Fam Med.

[36] Scolforo M AP News. Pennsylvania gives approval to first medical pot dispensary.

[37] Fedorova EV, Ataiants J, Wong CF, Iverson E, Lankenau SE (2022). Changes in medical cannabis patient status before and after cannabis legalization in California: associations with cannabis and other drug use. J Psychoactive Drugs.

[38] Levine M, Jontz A, Dabrowski P, Claudius IA, Kreisler R, Yee N, LoVecchio F (2021). Prevalence of marijuana use among trauma patients before and after legalization of medical marijuana: The Arizona experience. Subst Abus.

[39] Paschall MJ, Grube JW, Biglan A (2017). Medical marijuana legalization and marijuana use among youth in Oregon. J Prim Prev.

[40] Kondrad E, Reid A (2013). Colorado family physicians' attitudes toward medical marijuana. J Am Board Fam Med.

[41] Szyliowicz D, Hilsenrath P (2019). Medical marijuana knowledge and attitudes: a survey of the California Pharmacists Association. J Prim Care Community Health.

[42] Lombardi E, Gunter J, Tanner E (2020). Ohio physician attitudes toward medical Cannabis and Ohio's medical marijuana program. J Cannabis Res.

[43] Philpot LM, Ebbert JO, Hurt RT (2019). A survey of the attitudes, beliefs and knowledge about medical cannabis among primary care providers. BMC Fam Pract.

[44] Worster B, Ashare RL, Hajjar E, Garber G, Smith K, Kelly EL (2023). Clinician attitudes, training, and beliefs about cannabis: an interprofessional assessment. Cannabis Cannabinoid Res.

[45] Reinarman C, Nunberg H, Lanthier F, Heddleston T (2011). Who are medical marijuana patients? Population characteristics from nine California assessment clinics. J Psychoactive Drugs.

[46] Sideris A, Khan F, Boltunova A, Cuff G, Gharibo C, Doan LV (2018). New York physicians' perspectives and knowledge of the State Medical Marijuana Program. Cannabis Cannabinoid Res.

[47] Ryan J, Sharts-Hopko N (2017). The experiences of medical marijuana patients: a scoping review of the qualitative literature. J Neurosci Nurs.

[48] Pedersen W, Sandberg S (2013). The medicalisation of revolt: a sociological analysis of medical cannabis users. Sociol Health Illn.

[49] Bottorff JL, Bissell LJL, Balneaves LG, Oliffe JL, Capler NR, Buxton J (2013). Perceptions of cannabis as a stigmatized medicine: a qualitative descriptive study. Harm Reduct J.

[50] Satterlund TD, Lee JP, Moore RS (2015). Stigma among California's medical marijuana patients. J Psychoactive Drugs.

[51] Piper BJ, DeKeuster RM, Beals ML, Cobb CM, Burchman CA, Perkinson L, Lynn ST, Nichols SD, Abess AT (2017). Substitution of medical cannabis for pharmaceutical agents for pain, anxiety, and sleep. J Psychopharmacol.

[52] Heisey-Grove D, Rathert C, McClelland LE, Jackson K, DeShazo JP (2021). Patient and clinician characteristics associated with secure message content: retrospective cohort study. J Med Internet Res.

[53] El-Toukhy S, Méndez Alejandra, Collins S, Pérez-Stable Eliseo J (2020). Barriers to patient portal access and use: evidence from the Health Information National Trends Survey. J Am Board Fam Med.

[54] Armstrong K, Ravenell KL, McMurphy S, Putt M (2007). Racial/ethnic differences in physician distrust in the United States. Am J Public Health.

[55] Chang JC, Holland CL, Tarr JA, Rubio D, Rodriguez KL, Kraemer KL, Day N, Arnold RM (2017). Perinatal illicit drug and marijuana use. Am J Health Promot.

