# Supplementary material for: Discussions of Cannabis Over Patient Portal Secure Messaging: Content Analysis
Source: J Med Internet Res. 2024 Dec 12;26:e63311. doi: 10.2196/63311 (PMC11671783; doi:10.2196/63311)
Supplement: Multimedia Appendix 1 [file jmir_v26i1e63311_app1.docx]

Abbreviations: SDE (Smart Data Element); CUD (Cannabis Use Disorder); ICD (International Classification of Diseases); EDG (Eligibility Determination Group)

SDE List:

| SDE   - EPIC#18752 SocialHx_MJ_Freq - EPIC#21621 SocialHx_MJ_LastUse - EPIC#21622 SocialHx_MJ_MethodUse - GS#1001468 ProbList_CertProvider - GS#1001470 ProbList_AuthDispense - GS#1001471 ProbList_CertConditions - GS#1001473 ProbList_Dose - GS#1001479 ProbList_GeisingerDoc - GS#1001480 ProbList_DosageForm - GS#1001481 ProbList_ActiveIngredient - GS#1001482 ETX_Use |
| --- |

Lab List:

| LAB_LOINC_NM   - Tetrahydrocannabinol [Presence] in Urine by Screen method >50 ng/mL - Carboxy tetrahydrocannabinol [Mass/volume] in Serum or Plasma - Carboxy tetrahydrocannabinol [Mass/volume] in Serum or Plasma - Cannabinoids [Presence] in Urine by Screen method - Cannabinoids [Presence] in Urine by Screen method - Cannabinoids [Presence] in Urine by Screen method - Cannabinoids [Presence] in Urine by Screen method - Cannabinoids cutoff [Mass/volume] in Urine - Cannabinoids cutoff [Mass/volume] in Urine for Screen method - Cannabinoids confirm method [Identifier] in Urine - Cannabinoids [Mass/volume] in Urine by Confirmatory method - Cannabinoids [Mass/volume] in Urine by Confirmatory method - Carboxy tetrahydrocannabinol [Mass/volume] in Urine by Confirmatory method - Tetrahydrocannabinol [Presence] in Urine by Screen method >20 ng/mL - Tetrahydrocannabinol [Presence] in Urine by Screen method >100 ng/mL - Carboxy tetrahydrocannabinol [Mass/mass] in Meconium - Tetrahydrocannabinol [Presence] in Urine - Tetrahydrocannabinol [Mass/volume] in Serum or Plasma - Tetrahydrocannabinol [Mass/volume] in Urine - Cannabinoids [Presence] in Meconium by Confirmatory method - Tetrahydrocannabinol [Presence] in Meconium - Cannabinoids [Mass/volume] in Serum or Plasma - Cannabinoids [Presence] in Serum or Plasma by Screen method - Cannabinoids [Presence] in Serum or Plasma by Screen method |
| --- |

Medication List:

| med_nm  DRONABINOL  MARINOL 10 MG PO CAPS  DRONABINOL 10 MG PO CAPS  MARINOL 2.5 MG PO CAPS  DRONABINOL 2.5 MG PO CAPS  MARINOL 5 MG PO CAPS  DRONABINOL 5 MG PO CAPS  DRONABINOL 5 MG/ML PO SOLN  SYNDROS 5 MG/ML PO SOLN  NABILONE  CESAMET 1 MG PO CAPS  NABILONE 1 MG PO CAPS  CANNABIDIOL (ANTICONVULSANTS - MISC.)  CANNABIDIOL 100 MG/ML ORAL LIQUID STUDY  CANNABIDIOL 100 MG/ML PO SOLN  EPIDIOLEX 100 MG/ML PO SOLN  CANNABINOIDS  FULL SPECTRUM SOFT GELS 15 MG PO CAPS  FULL SPECTRUM SALVE 5 MG/GM EX OINT  FULL SPECTRUM EXTRACT 10 MG/ML PO LIQD  FULL SPECTRUM EXTRACT 20 MG/ML PO LIQD  THC FREE 20 MG/ML PO LIQD | med_generic_nm  DRONABINOL  DRONABINOL CAP 10 MG  DRONABINOL CAP 10 MG  DRONABINOL CAP 2.5 MG  DRONABINOL CAP 2.5 MG  DRONABINOL CAP 5 MG  DRONABINOL CAP 5 MG  DRONABINOL SOLN 5 MG/ML  DRONABINOL SOLN 5 MG/ML  NABILONE  NABILONE CAP 1 MG  NABILONE CAP 1 MG  CANNABIDIOL  CANNABIDIOL  CANNABIDIOL SOLN 100 MG/ML  CANNABIDIOL SOLN 100 MG/ML  CANNABINOIDS  CANNABINOIDS CAP 15 MG  CANNABINOIDS OINTMENT 5 MG/GM  CANNABINOIDS ORAL LIQUID 10 MG/ML  CANNABINOIDS ORAL LIQUID 20 MG/ML  CANNABINOIDS ORAL LIQUID 20 MG/ML |
| --- | --- |
