# Supplementary material for: Discussions of Cannabis Over Patient Portal Secure Messaging: Content Analysis
Source: J Med Internet Res. 2024 Dec 12;26:e63311. doi: 10.2196/63311 (PMC11671783; doi:10.2196/63311)
Supplement: Multimedia Appendix 2 [file jmir_v26i1e63311_app2.docx]

Terms from initial cannabis lexicon

“Cannabis”

1) 'marijuana': 0.81

2) 'thc': 0.74

3) 'edible': 0.72

4) 'medicinal': 0.70

5) 'tinctures': 0.68

6) 'violates': 0.68

7) 'illegal': 0.68

8) 'vape': 0.67

9) 'marajuana': 0.67

10) 'illicit': 0.67

“Cannabidiol”

No generated list

“Cannabinoid”

1)'hyperemesis': 0.67

2) 'cannabis': 0.65

3) 'concomitant': 0.64

4)'hyperlipidemia': 0.62

5) 'bisphosphonates': 0.62

6) 'psychotropic': 0.62

7) 'terpenes': 0.61

8) 'conventional': 0.61

9) 'chemotherapies': 0.61

10) 'marijuana': 0.61

“Cannabinol”

1) 'nuerotin': 0.74

2) 'cymbolta': 0.73

3) 'benzodiazapines': 0.71

4) 'agonists': 0.70

5) 'metabolizes': 0.70

6) 'nly': 0.69

7)'pottasium': 0.69

8) 'toprimate': 0.69

9) 'depressions': 0.68

10) 'antidepressent': 0.68

“Cannabig”

No generated list

“Cannabaceae”

No generated list

“Cannabinaceae”

No generated list

“Cannabinum”

No generated list

“Cbd”

1) 'thc': 0.76

2) 'tincture': 0.74

3) 'tinctures': 0.74

4) 'oil': 0.73

5) 'edibles': 0.72

6) 'oils': 0.72

7) 'ilera': 0.72

8) 'mmj': 0.70

9) 'hemp': 0.70

10) 'sativa': 0.68

“Cbg”

1) 'decaffeinated': 0.62

2) 'carpe': 0.61

3) 'waffles:, 0.61

4)'fig': 0.60

5) 'estroven': 0.60

6) 'coco': 0.60

7) 'oreos': 0.60

8) 'teas': 0.60

9) 'grilled': 0.60

10) 'amino': 0.60

“Cbn”

1) 'cnrn': 0.87

2) 'cnsc': 0.85

3) 'neurotrauma': 0.82

4) 'neu': 0.81

5) 'kboudeman': 0.79

6) 'cdces': 0.79

7) 'rosciences': 0.79

8) 'foregut': 0.79

9) 'cmsrn': 0.78

10) 'cnor': 0.77

“Ganga”

No generated list

“Ganja”

No generated list

“Hash”

1) 'utm_campaign': 0.71

2) 'fpain': 0.69

3) utm_medium': 0.67

4) 'yw': 0.67

5) 'picprop': 0.67

6) 'coo': 0.67

7) 'oficina': 0.67

8) 'dol': 0.67

9) 'farinae': 0.67

10) 'url': 0.66

“Hasheesh”

No generated list

“Hashesh”

No generated list

“Hashish”

No generated list

“Hemp”

1) 'oil': 0.78

2) ‘cbd': 0.70

3) 'primrose': 0.69

4) 'coconut': 0.67

5) 'ilera': 0.67

6) 'oils': 0.67

7) 'gummies': 0.66

8) 'herbals': 0.66

9) 'castor': 0.66,

10) 'chondroitin': 0.65

“Indica”

1) 'sativa': 0.77

2) 'ilera': 0.75

3) 'flower': 0.74,

4) 'hybrid': 0.74

5) 'extracts': 0.73

6) 'tinctures': 0.73

7) 'neti': 0.71

8) 'squirt': 0.71

9) 'vapo': 0.69

10) 'arjuna': 0.69

“Kef”

No generated list

“Keif”

No generated list

“Marijuana”

1) 'cannabis': 0.81

2) 'medicinal': 0.80

3) 'marajuana': 0.78

4) 'mmj': 0.73

5) 'card': 0.70

6) 'tinctures': 0.69

7) 'medical': 0.69

8) 'federally': 0.67

9) 'cbd': 0.67

10) 'thc': 0.66

“Mj”

1) 'marijuana': 0.62

2) 'marj': 0.60

3) 'mmj': 0.56

4) 'marajuana': 0.56

5) 'card': 0.55

6) '[Name]': 0.54

7) 'marij': 0.53

8) '[Name]': 0.53

9) 'certifications': 0.53

10) '[Name]': 0.53

“Mjx”

No generated list

“Mmj”

1) 'marijuana': 0.73

2) 'cbd': 0.70

3) 'tinctures': 0.66

4) 'edibles': 0.65

5) 'marajuana': 0.63

6) 'tincture': 0.61

7) 'medicinal': 0.61

8) 'ilera': 0.60

9) 'marj': 0.60

10) 'cannabis': 0.60

“Mmjx”

No generated list

“Mootah”

No generated list

“Pot”

1) 'netti': 0.69

2) 'netty': 0.69

3) 'neti': 0.66

4) ‘vapor’: 0.65

5) ‘oxymetazoline’: 0.61

6) 'saltwater’': 0.61

7) ‘netting’: 0.60

8) 'gels': 0.60

9) 'vaporub': 0.60

10) 'ayr': 0.59

“Pot” (excluded messages w/ neti pot, netti pot, netty pot, netting pot)

1) 'brownie': 0.61

2) 'meth': 0.59

3) 'dope': 0.59

4) 'smoked': 0.59

5) 'hemp': 0.59

6) 'kratom': 0.58

7) 'artifical': 0.57

8) 'narc': 0.57

9) 'marijuana': 0.57

10) ‘weed’: 0.56

“Pothead”

No generated list

“Reefer”

No generated list

“Ruderalis”

No generated list

“Sativa”

1) 'hybrid': 0.79

2) 'indica': 0.77

3) 'ilera': 0.76

4) 'rso': 0.75

5) 'extracts': 0.74

6) 'flower': 0.73

7) 'veggie': 0.72

8) 'irest': 0.72

9) 'arjuna': 0.71

10) 'cayenne': 0.71

“Sinsemilla”

No generated list

“Spliff”

No generated list

“Tetrahydrocannabinol”

No generated list

“Thc”

1) 'cbd': 0.76

2) 'cannabis': 0.74

3) 'methamphetamine': 0.68

4) 'cannabinoids': 0.68

5) 'violates': 0.66

6) 'marijuana': 0.66

7) 'tinctures': 0.65

8) 'edibles': 0.65

9) 'amphetamines': 0.62

10) 'weed': 0.62

“Weed”

1) 'dope': 0.68

2) 'tinctures': 0.66

3) 'brownie': 0.64

4) 'cannabis': 0.64

5) 'edible': 0.63

6) 'laced': 0.63

7) 'thc': 0.62

8) 'opium': 0.62

9) 'smoking': 0.61

10) 'ilera': 0.60

“Zut”

No generated list

Terms added from initial lexicon similarity lists

“Edible”

1) 'cannabis': 0.72

2) 'tinctures': 0.71

3) 'speeding': 0.69

4) 'marketing': 0.67

5) 'edibles': 0.66

6) 'recreationally': 0.66

7) 'brownie': 0.66

8) 'browsing': 0.65

9) 'indica': 0.65

10) 'tincture': 0.65

“Edibles”

1) 'cbd': 0.72

2) 'tinctures': 0.71

3) 'ilera': 0.71

4) 'oil': 0.68

5) 'edible': 0.66

6) 'sativa': 0.65

7) 'thc': 0.65

8) 'mmj': 0.65

9) 'rso': 0.65

10) 'cannabis': 0.64

“Ilera”

1) 'sativa': 0.76

2) 'indica': 0.75

3) 'tinctures': 0.75

4) 'tincture': 0.74

5) 'cbd': 0.72

6) 'shine': 0.71

7) 'flower': 0.71,

8) 'lavender': 0.71

9) 'edibles': 0.71

10) 'clove': 0.70

“Marj”

1) 'marjuana': 0.68

2) 'tinctures': 0.68

3) 'marajuana': 0.67

4) 'professions': 0.65

5) 'fiorocet': 0.65

6) 'nueropathy': 0.64

7) 'marij': 0.64

8) 'marijuana': 0.63

9) 'certifications': 0.63

10) 'federally': 0.63

“Marjuana”

1) 'marj': 0.68

2) 'recreationally': 0.67

3) 'rational': 0.64

4) 'soclean': 0.63

5) 'edible': 0.62

6) 'mariajuana': 0.62

7) 'marijuanna': 0.62

8) 'tinctures': 0.61

9) 'thermacare': 0.60

10) 'condone': 0.60

“Marijuanna”

1) 'violates': 0.66

2) 'marijuana': 0.66

3) 'mariajuana': 0.66

4) 'cannabis': 0.64

5) 'mmwr': 0.64

6) 'federally': 0.64

7) 'justifying': 0.64

8) 'illicit': 0.64

9) 'marihuana': 0.63

10) 'edible': 0.63

“Mariajuana”

1) 'federally': 0.69

2) 'violates': 0.68

3) 'marijuana': 0.66

4) 'marihuana': 0.66

5) 'marijuanna': 0.66

6) 'prescribers': 0.66

7) 'pharmacological': 0.66

8) 'rational': 0.65

9) 'certifying': 0.65

10) 'bias': 0.65

“Marihuana”

1) 'certifications': 0.74

2) 'federally': 0.67

3) 'mariajuana': 0.66

4) 'parental': 0.63

5) 'iate': 0.63

6) 'marijuanna': 0.63

7) 'marijuana': 0.63,

8) 'nccn': 0.62

9) 'certifying': 0.62

10) 'healt': 0.62

“Tincture”

1) 'cbd': 0.74

2) 'ilera': 0.74

3) 'edible': 0.65

4) 'tinctures': 0.64

5) 'indica': 0.64

6) 'oil': 0.63

7) 'castor': 0.63

8) 'edibles': 0.62

9) 'sativa': 0.61

10) 'mmj': 0.61

“Tinctures”

1) 'ilera': 0.75

2) 'cbd': 0.74

3) 'indica': 0.73

4) 'edible': 0.71

5) 'edibles': 0.71

6) 'sativa': 0.70

7) 'marijuana': 0.69

8) 'cannabis': 0.68

9) 'marj': 0.69

10) 'oils': 0.67
