# Supplementary material for: Discussions of Cannabis Over Patient Portal Secure Messaging: Content Analysis
Source: J Med Internet Res. 2024 Dec 12;26:e63311. doi: 10.2196/63311 (PMC11671783; doi:10.2196/63311)
Supplement: Multimedia Appendix 3 [file jmir_v26i1e63311_app3.docx]

Patient Message Codebook

| Code Category | | Definition |
| --- | --- | --- |
|  | |  |
| **1 Correctly classified message** | | Use these codes to indicate whether the message includes a mention of plant-based marijuana |
|  | 1.1 Yes | Message includes a mention of plant-based marijuana |
|  | 1.2 No, not related to marijuana | Message does not include a mention of plant-based marijuana or any cannabis-related product |
|  | 1.3 No, reference to Rx | Message does not include a mention of plant-based marijuana, but includes a mention of a cannabinoid-derived Rx |
| **2 Message author** | | Use these codes to indicate whether it appears that the message was written by the patient, or someone else on behalf of the patient |
|  | 2.1 Patient | Message appears to be written by the patient |
|  | 2.2 Non-patient | Message appears to be written on behalf of the patient by someone else |
| **3 Time of use** | | Use these codes to indicate the timing of the patient's use of marijuana |
|  | 3.1 Past, but not current | Patient used marijuana in the past, but does not currently use |
|  | 3.2 Current | Patient currently uses marijuana |
|  | 3.3 Interest/intent to use | Patient has an interest in using or intends to use marijuana |
|  | 3.4 None | None of the above categories apply |
| **4 Reason for use** | | Use these codes to indicate the specific named health issue that the patient has used, is using, or wants to use cannabis for |
|  | 4.1 Unspecified | A specific issue is not stated. |
|  | 4.2 Appetite | The stated reason for patient use is appetite issues |
|  | 4.3 Nausea | The stated reason for patient use is nausea |
|  | 4.4 Pain | The stated reason for patient use is pain |
|  | 4.4.1 Pain (unspecified) | The stated reason for patient use is pain (unspecified) |
|  | 4.4.2 Back pain | The stated reason for patient use is back pain, problems, or issues |
|  | 4.4.3 Hip pain | The stated reason for patient use is hip pain, problems, or issues |
|  | 4.4.4 Joint pain | The stated reason for patient use is joint pain, problems, or issues |
|  | 4.4.5 Neck pain | The stated reason for patient use is neck pain, problems, or issues |
|  | 4.4.6 Knee pain | The stated reason for patient use is knee pain, problems, or issues |
|  | 4.4.7 Shoulder pain | The stated reason for patient use is shoulder pain, problems, or issues |
|  | 4.4.8 Foot pain | The stated reason for patient use is feet pain, problems, or issues |
|  | 4.5 Glaucoma | The stated reason for patient use is glaucoma |
|  | 4.6 Skin irritation | The stated reason for patient use is skin irritation |
|  | 4.7 Sleep | The stated reason for patient use is to help with sleep |
|  | 4.8 Anxiety | The stated reason for patient use is anxiety |
|  | 4.9 Mood swings | The stated reason for patient use is mood swings |
|  | 4.10 Migraine | The stated reason for patient use is migraines or headaches |
|  | 4.11 Post traumatic stress disorder (PTSD) | The stated reason for patient use is PTSD or c-PTSD |
|  | 4.12 Inflammation | The stated reason for patient use is inflammation |
|  | 4.13 Fibromyalgia | The stated reason for patient use is fibromyalgia |
|  | 4.14 Endometrial | The stated reason for patient use is endometrial |
|  | 4.15 Cramps | The stated reason for patient use is cramps |
|  | 4.16 Spasms | The stated reason for patient use is spasms |
|  | 4.17 Periods | The stated reason for patient use is period symptoms |
|  | 4.18 Racing thoughts | The stated reason for patient use is to manage/control racing thoughts |
|  | 4.19 Asthma | The stated reason for patient use is asthma |
|  | 4.20 Vertigo | The stated reason for patient use is vertigo |
|  | 4.21 Primary ciliary dyskinesia (PCD) | The stated reason for patient use is PCD |
|  | 4.22 Hepatitis C | The stated reason for patient use is for hepatitis C |
|  | 4.23 Seizure | The stated reason for patient use is seizures |
|  | 4.24 Opioid use | The stated reason for patient use is opioid use |
|  | 4.25 Tremors | The stated reason for patient use is tremors |
|  | 4.26 Attention deficit disorder (ADHD) | The stated reason for patient use is ADHD |
|  | 4.27 Depression | The stated reason for patient use is depression |
|  | 4.28 Inflammatory bowel syndrome (IBS) | The stated reason for patient use is IBS |
|  | 4.29 Manic episodes | The stated reason for patient use is manic episodes |
| **5 Purpose of cannabis mention** | | Use these codes to indicate the primary reasons why the patient (or person on behalf of patient) is discussing marijuana |
|  | 5.1 Seeking guidance related to use | Patient seeking guidance related to marijuana use |
|  | 5.1.1 Seeking guidance - Recommendation to use | Patient asking if the provider would recommend marijuana use for a health issue |
|  | 5.1.2 Seeking guidance - Modify use | Patient asking the provider if they should increase, decrease, or stop use of marijuana |
|  | 5.1.3 Seeking guidance - Adverse event | Patient asking the provider if using marijuana is causing or would cause an adverse event |
|  | 5.1.4 Seeking guidance - Drug interaction | Patient asking the provider if using marijuana would cause a drug-to-drug interaction |
|  | 5.2 Seeking assistance related to use | Patient seeking assistance related to use of marijuana |
|  | 5.2.1 Assistance - Prescription | Patient asking if the provider does or can prescribe medical marijuana (including a medical marijuana card |
|  | 5.2.2 - Assistance - Referral | Patient asking if the provider can give a referral to another provider who can help with medical marijuana/marijuana card access |
|  | 5.2.3 Assistance - Insurance coverage | Patient is asking if the provider can assist in getting insurance to cover medical marijuana |
|  | 5.2.4 Assistance - Request for formal approval to use | Patient is asking the provider for formal approval to use marijuana, such as a letter or other documentation. This includes asking for a formal diagnosis stating that the patient has a marijuana qualifying condition |
|  | 5.2.5 Assistance - Access | Patient is asking the provider for assistance in getting first time access to medical marijuana/marijuana card, but is not specifically asking for a prescription, referral, help with insurance coverage, or formal documentation |
|  | 5.3 Discussion of marijuana screening results | Patient discussing the results of a screening for marijuana use |
|  | 5.3.1 Positive result - Denies use or excessive use | Patient received a positive result or fears a positive result due to previous use, but denies any recent use or excessive use. |
|  | 5.3.2 Positive result - Reputational concern | Patient concerned about their reputation among providers after positive result. |
|  | 5.3.3 Positive result - Restricted medication or treatment access concern | Patient concerned about restricted medication or treatment access after positive result. |
|  | 5.3.4 Positive result - Lack of knowledge | Patient received a positive result and claims they did not know that the substance they took would flag as positive. |
|  | 5.3.5 Request - Order for screening | Patient requesting an order for a screening or results of recent screening to prove no current use. |
|  | 5.3.6 Thanking for screening results | Patient thanks provider for sending screening results. |
|  | 5.3.7 Evidence of negative result | Patient informing provider that another provider has sent/will send screening results showing that patient is no longer using |
|  | 5.3.8 Anticipating positive result | Patient anticipates a positive result on screening due to current use |
|  | 5.4 Expression of displeasure over stigmatization related to use | Patient expressing displeasure over perceived stigmatization related to marijuana use. |
|  | 5.5 Explanation of previous use | Patient explaining the circumstances around previous use of marijuana |
|  | 5.5.1 Used in the past to treat health issue | Patient explaining that they used marijuana in the past to treat a health-related issue |
|  | 5.5.2 Experienced negative outcome due to past use | Patient explaining that they perceived negative outcomes (health or non-health) from past marijuana use |
|  | 5.5.3 Used in the past for recreational purpose | Patient explaining that they used marijuana in the past for recreational purpose |
|  | 5.5.4 Accidental Use | Patient explaining that they used marijuana in the past, but that it was accidental |
|  | 5.6 Report of current use status | Patient explicitly reporting the current status of their marijuana use |
|  | 5.6.1 Currently using - Specified purpose | Patient stating that they are currently using marijuana and describes reason for using |
|  | 5.6.2 Currently using - Unspecified purpose | Patient currently using marijuana, but doesn't state what use is for |
|  | 5.6.3 Currently using - With another substance/prescription | Patient mentions current marijuana use in the context of discussing use of other substances or prescriptions |
|  | 5.6.4 Currently using - Side effects | Patient currently using marijuana and is describing perceived side effects |
|  | 5.6.5 Currently using - Desires to stop use | Patient currently using marijuana, but desires to stop use |
|  | 5.6.6 Currently using - Agrees to stop or reduce use | Patient currently using marijuana, but agrees to stop or reduce use |
|  | 5.6.7 Currently using - Perceived negative outcome from non-use | Patient currently using marijuana and reports perceived negative outcomes (health or non-health) during times of non-use |
|  | 5.6.8 Currently using - Ineffective or inconsistent | Patient currently using marijuana, but perceives ineffectiveness or inconsistency in helping with health issue |
|  | 5.6.9 Not currently using - Ineffective or inconsistent | Patient not currently using because of perceived ineffectiveness or inconsistency in helping with health issue |
|  | 5.6.10 Not currently using - Perceived negative outcome from use | Patient not currently using marijuana and reports perceived negative outcomes (health or non-health) associated with use |
|  | 5.6.10 Not currently using - Medication/treatment access | Patient not currently using marijuana in order to receive access to medication/treatment |
|  | 5.6.11 Not currently using - Employment | Patient not currently using marijuana for reasons related to employment |
|  | 5.6.12 Not currently using - Unspecified purpose | Patient not currently using marijuana and the reason for non-use is unspecified |
|  | 5.6.13 Not currently using - Current condition | Patient not currently using marijuana due to a medical condition |
|  | 5.7 Complaining about marijuana | Patient complaining about issue related to marijuana |
|  | 5.7.1 Complaint - Not related to own use | Patient making general complaints about marijuana, not related to own use |
|  | 5.7.2 Complaint - Medical marijuana access process | Patient complaining about the process of getting access to medical marijuana |
|  | 5.8 Not enough context to determine purpose | Message does not contain enough information to determine purpose of mention |
|  | 5.9 Cost Prohibitive | Patient mentions marijuana is too expensive |
|  | 5.10 Request to change marijuana mention in EHR notes | Patient requests that their marijuana use status be changed (removed or added) |
|  | 5.11 Resources for MMJ | Patient informing provider about marijuana |
|  | 5.11.1 Research/Studies | Patient informing provider about research/studies on marijuana |
|  | 5.11.2 Personal anecdote | Patient informing provider about marijuana benefits or harms based on personal anecdotes |
|  | 5.12 Patient-related MMJ documentation | Patient providing personal marijuana related documentation |
|  | 5.12.1 Perceived need for integration of care | Patient providing personal marijuana related documentation for perceived need for integration of care. Patient perceives provider needs marijuana related information that happened with another provider, so patient attaches or provides information in the message |
|  | 5.12.2 Proof of qualification | Patient providing personal marijuana documentation as proof of qualification to use |
|  | 5.13 Acknowledgement of provider perspective | Patient acknowledges provider perspective on marijuana |
|  | 5.14 Case management for medical marijuana | Patient states that they are getting/seeking help from case management for marijuana use |
|  | 5.15 Statement of plan to use | Patient makes a statement of their plan to use marijuana. This may include a description of the plan to use. |
|  | 5.15.1 Statement of plan - Use with pain medication | Patient states that they plan to use marijuana with pain medication |
|  | 5.15.2 Statement of plan - Substitute for pain medication | Patient states that they plan to use marijuana as a substitute for pain medication |
|  | 5.15.3 Statement of plan - Approved to use | Patient states that they have been approved to use marijuana |
|  | 5.15.4 Statement of plan - Ordered product | Patient states that they have ordered a marijuana product |
|  | 5.15.5 Statement of plan - Certification | Patient states that they are planning to get a medical marijuana certification |
|  | 5.15.6 Statement of plan - Substitute for anxiety medication | Patient states that they plan to use marijuana as a substitute for anxiety medication |
|  | 5.16 Debating use | Patient informs provider that they are debating whether or not to use marijuana |
|  | 5.17 Statement of preference over medications | Patient states that they would prefer to use marijuana over medications |

Provider Message Codebook

| **Code Category** | | **Definition** |
| --- | --- | --- |
|  | |  |
| **1 Correctly classified message** | | Use these codes to indicate whether the message includes a mention of plant-based marijuana |
|  | 1.1 Yes | Message includes a mention of plant-based marijuana |
|  | 1.2 No, not related to marijuana | Message does not include a mention of plant-based marijuana or any cannabis-related product |
|  | 1.3 No, reference to Rx | Message does not include a mention of plant-based marijuana, but includes an mention of a cannabinoid-derived Rx |
| **2 Message recipient** | | Use these codes to indicate whether it appears that the message was written to the patient, or someone else in place of the patient |
|  | 2.1 Patient | Message appears to be written to the patient |
|  | 2.2 Non-patient | Message appears to be written to someone else in place of the patient |
| **3 Reason for use** | | Use these codes to indicate the specific named health issue that the patient has used, is using, or wants to use cannabis for, as stated by the provider |
|  | 3.1 Unspecified | A specific issue is not stated |
|  | 3.2 Pain | The stated reason for patient use is to manage pain |
|  | 3.2.1 Pain (unspecified) | The stated reason for patient use is to manage pain (unspecified) |
|  | 3.2.2 Neck Pain | The stated reason for patient use is neck pain, problems, or issues |
|  | 3.2.3 Chronic Refractory Pain | The stated reason for patient use is chronic refractory pain |
|  | 3.3 Sleep | The stated reason for patient use is for sleep |
|  | 3.4 Anxiety | The stated reason for patient use is for anxiety |
|  | 3.5 Inflamed skin issues | The stated reason for patient use is for inflammed skin issues |
|  | 3.6 Seizure | The stated reason for patient use is for seizures |
|  | 3.7 Inflammation | The stated reason for patient use is inflammation |
|  | 3.8 Headaches | The stated reason for patient use is headaches |
|  | 3.9 Glioblastoma | The stated reason for patient use is glioblastoma |
| **4 Purpose of cannabis mention** | | Use these codes to indicate the primary reasons why the provider is discussing marijuana |
|  | 4.1 Provide guidance on patient use | Provider giving guidance to patient related to use of marijuana |
|  | 4.1.1 Recommendation - Use marijuana | Provider recommending that patient start or continue using marijuana |
|  | 4.1.2 Recommendation - Stop using marijuana | Provider recommending that patient stop or reduce frequency/dosage of marijuana |
|  | 4.1.3 Explanation - Positive effects of use | Provider explaining/educating patient on the positive effects of using marijuana |
|  | 4.1.4 Assistance - Medical marijuana access | Provider assisting or providing guidance to patient on accessing medical marijuana |
|  | 4.1.5 Explanation - Drug Interaction | Provider explaining to patient about possible interaction between marijuana and another drug (either that there is an interaction or no interaction) |
|  | 4.1.6 Explanation - Negative effects of use | Provider explaining/educating patient on the negative effects of using marijuana, not including negative effects related to a drug-drug interaction |
|  | 4.1.7 Explanation - Insurance coverage | Provider explaining circumstance around insurance coverage of marijuana to patient |
|  | 4.1.8 Recommendation - Do not use | Provider recommending that patient not use marijuana due to clinical risk |
|  | 4.1.9 Recommendation - Time of use | Provider making recommendation around the timing of marijuana use |
|  | 4.1.10 Explanation - No effect of use | Provider explaining to patient that marijuana has no effect on patient's current condition or complaint |
|  | 4.2 Unable to refer, prescribe, or recommend use | Provider stating or implying that they are unable to refer, prescribe, or recommend use of marijuana |
|  | 4.2.1 Legal | Provider is unable to recommend for legal reasons |
|  | 4.2.2 Geisinger policy | Provider is unable to recommend due to Geisinger policy |
|  | 4.2.3 Outside scope | Provider is unable to recommend because it is not within the scope of their work or practice |
|  | 4.2.4 Unspecified | Provider is unable to recommend for an unclear or unspecified reason |
|  | 4.2.5 Lack of evidence for efficacy | Provider is unable to recommend due to lack of evidence on the efficacy of marijuana |
|  | 4.2.6 Unavailability | Provider is unable to recommend due to unavailability of medical marijuana |
|  | 4.3 Provide information on marijuana/cannabis screening | Provider giving information related to urine toxicology screening |
|  | 4.3.1 Inform - Screening ordered | Provider informing patient that a screening has been ordered |
|  | 4.3.2 Inform - Positive result | Provider informing patient of a positive screening result |
|  | 4.3.3 Inform - Medication use agreement violation | Provider informing patient that they (patient) have violated MUA and/or provider informs they cannot be prescribe medication due to positive screening result |
|  | 4.3.4 Inform - Educating about screening | Provider educating patient about the purpose of screening |
|  | 4.4 Inquiry about patient use | Provider inquiring about patient’s use of marijuana |
|  | 4.4.1 Inquiry - Use or exposure | Provider asking if patient has been using or has been exposed to marijuana |
|  | 4.4.2 Inquiry - Stopped using | Provider asking if patient has stopped or will stop using marijuana |
|  | 4.4.3 Inquiry - Effects of use | Provider asking patient how marijuana is affecting the patient (beneficial, harmful, or neutral) |
|  | 4.4.4 Inquiry - Appointment | Provider asking if patient has had or will have an appointment with another provider about using marijuana |
|  | 4.4.5 Inquiry - Reason for use | Provider asking patient the reason why they have or are using marijuana |
|  | 4.4.6 Inquiry - Dosage | Provider asking patient for the size and/or frequency of dose of THC and/or CBD in product |
|  | 4.5 Provider-to-Provider Consideration | Provider informing patient that they have or will consult another provider about patient's marijuana use, or they are asking about another provider's recommendations |
|  | 4.6 Mention of current use status | Provider mentions that patient is using/not using marijuana |
|  | 4.7 Statement of uncertainty | Provider states they are unsure on their view or lack knowledge about marijuana |
|  | 4.8 Research on medical marijuana | Provider discussing research related to medical marijuana |
|  | 4.8.1 Inform on current state of research | Provider is informing patient about the current state of medical marijuana research |
|  | 4.8.2 Suggestion to self-educate | Provider suggests that patient research medical marijuana on their own |
|  | 4.8.3 Plan to self-education | Provider informing patient that they plan to educate themselves on the research related to medical marijuana |
|  | 4.9 Request to see medical marijuana card | Provider requesting patient to bring/show medical marijuana card |
