# Supplementary figures and images for: Discussions of Cannabis Over Patient Portal Secure Messaging: Content Analysis
Source: J Med Internet Res. 2024 Dec 12;26:e63311. doi: 10.2196/63311 (PMC11671783; doi:10.2196/63311)

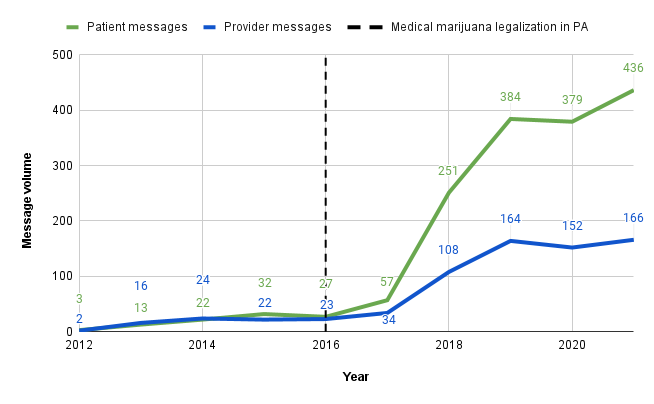

Supplement: Multimedia Appendix 4 [file jmir_v26i1e63311_app4.png]

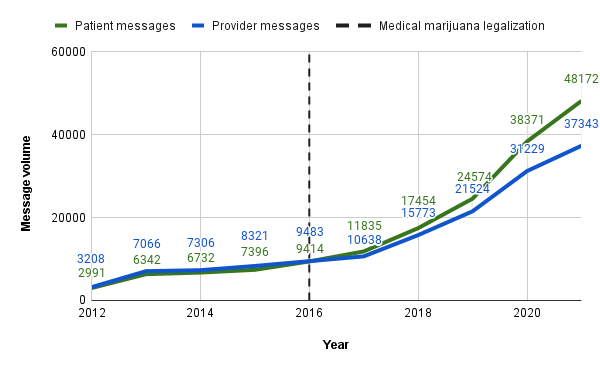

Supplement: Multimedia Appendix 5 [file jmir_v26i1e63311_app5.png]
