# Supplementary material for: Discussions of Cannabis Over Patient Portal Secure Messaging: Content Analysis
Source: J Med Internet Res. 2024 Dec 12;26:e63311. doi: 10.2196/63311 (PMC11671783; doi:10.2196/63311)
Supplement: Multimedia Appendix 6 [file jmir_v26i1e63311_app6.docx]

Patient code frequencies

| Content Category | | 2012-2016 (n=20), n (%) | 2017-2022 (n=170), n (%) |
| --- | --- | --- | --- |
|  | |  |  |
| **1 Correctly classified message** | |  |  |
|  | 1.1 Yes | 18 (90.0) | 162 (95.3) |
|  | 1.2 No, not related to marijuana | 1 (5.0) | 8 (4.7) |
|  | 1.3 No, reference to Rx | 1 (5.0) | - |
| **2 Message author** | |  |  |
|  | 2.1 Patient | 17 (94.4) | 153 (94.4) |
|  | 2.2 Non-patient | 1 (5.6) | 9 (5.6) |
| **3 Time of use** | |  |  |
|  | 3.1 Past, but not current | 5 (27.8) | 18 (11.1) |
|  | 3.2 Current | 5 (27.8) | 53 (32.7) |
|  | 3.3 Interest/intent to use | 5 (27.8) | 80 (49.4) |
|  | 3.4 None | 3 (16.7) | 18 (11.1) |
| **4 Reason for use** | |  |  |
|  | 4.1 Unspecified | 9 (50.0) | 70 (43.2) |
|  | 4.2 Appetite | - | 5 (3.1) |
|  | 4.3 Nausea | 1 (5.6) | 2 (1.2) |
|  | 4.4 Pain | 8 (44.4) | 70 (43.2) |
|  | 4.4.1 Pain (unspecified) | 7 (38.9) | 49 (30.2) |
|  | 4.4.1 Back pain | 1 (5.6) | 9 (5.6) |
|  | 4.4.2 Hip pain | - | 3 (1.9) |
|  | 4.4.3 Joint pain | - | 2 (1.2) |
|  | 4.4.4 Neck pain | 1 (5.6) | 2 (1.2 |
|  | 4.4.5 Knee pain | - | 1 (0.6) |
|  | 4.4.6 Shoulder pain | - | 1 (0.6) |
|  | 4.4.7 Foot pain | - | 3 (1.9) |
|  | 4.5 Skin irritation | - | 1 (0.6) |
|  | 4.6 Sleep | - | 21 (13) |
|  | 4.7 Anxiety | 1 (5.6) | 25 (15.4) |
|  | 4.8 Mood swings | - | 1 (0.6) |
|  | 4.9 Migraine | 3 (16.7) | 6 (3.7) |
|  | 4.10 Post traumatic stress disorder (PTSD) | 1 (5.6) | 5 (3.1) |
|  | 4.11 Inflammation | - | 2 (1.2) |
|  | 4.12 Fibromyalgia | - | 8 (4.9) |
|  | 4.13 Endometrial | - | 1 (0.6) |
|  | 4.14 Cramps | - | 1 (0.6) |
|  | 4.15 Spasms | - | 2 (1.2) |
|  | 4.16 Periods | - | 1 (0.6) |
|  | 4.17 Racing thoughts | - | 1 (0.6) |
|  | 4.18 Asthma | - | 1 (0.6) |
|  | 4.19 Vertigo | - | 1 (0.6) |
|  | 4.20 Primary ciliary dyskinesia (PCD) | - | 1 (0.6) |
|  | 4.21 Hepatitis C | - | 1 (0.6) |
|  | 4.22 Seizure | - | 1 (0.6) |
|  | 4.23 Opioid use | - | 1 (0.6) |
|  | 4.24 Tremors | - | 1 (0.6) |
|  | 4.25 Attention deficit disorder (ADHD) | - | 1 (0.6) |
|  | 4.26 Depression | - | 4 (2.5) |
|  | 4.27 Inflammatory bowel syndrome (IBS) | - | 1 (0.6) |
|  | 4.28 Manic episodes | - | 1 (0.6) |
| **5 Purpose of cannabis mention** | |  |  |
|  | 5.1 Seeking guidance related to use | 1 (5.6) | 36 (22.2) |
|  | 5.1.1 Seeking guidance - Recommendation to use | 1 (5.6) | 23 (14.2) |
|  | 5.1.2 Seeking guidance - Modify use | - | 3 (1.9) |
|  | 5.1.3 Seeking guidance - Adverse event | - | 5 (3.1) |
|  | 5.1.4 Seeking guidance - Drug interaction | - | 6 (3.7) |
|  | 5.2 Seeking assistance related to use | 4 (22.2) | 57 (35.2) |
|  | 5.2.1 Assistance - Prescription | 1 (5.6) | 6 (3.7) |
|  | 5.2.2 - Assistance - Referral | - | 13 (8.0) |
|  | 5.2.3 Assistance - Insurance coverage | 1 (5.6) | - |
|  | 5.2.4 Assistance - Request for formal approval to use | 1 (5.6) | 32 (19.8) |
|  | 5.2.5 Assistance - Access | 1 (5.6) | 9 (5.6) |
|  | 5.3 Discussion of marijuana screening results | 7 (38.9) | 9 (5.6) |
|  | 5.3.1 Positive result - Denies use or excessive use | 3 (16.7) | 2 (1.2) |
|  | 5.3.2 Positive result - Reputational concern | 1 (5.6) | - |
|  | 5.3.3 Positive result - Restricted medication or treatment access concern | 4 (22.2) | 3 (1.9) |
|  | 5.3.4 Positive result - Lack of knowledge | - | 2 (1.2) |
|  | 5.3.5 Request - Order for screening | - | 1 (0.6) |
|  | 5.3.6 Thanking for screening results | - | 1 (0.6) |
|  | 5.3.7 Evidence of negative result | 1 (5.6) | - |
|  | 5.3.8 Anticipating positive result | - | 1 (0.6) |
|  | 5.4 Expression of displeasure over stigmatization related to use | - | 2 (1.2) |
|  | 5.5 Explanation of previous use | 2 (11.1) | 13 (8.0) |
|  | 5.5.1 Used in the past to treat health issue | 2 (11.1) | 8 (4.9) |
|  | 5.5.2 Experienced negative outcome due to past use | - | 2 (1.2) |
|  | 5.5.3 Used in the past for recreational purpose | - | 2 (1.2) |
|  | 5.5.4 Accidental Use | - | 1 (0.6) |
|  | 5.6 Report of current use status | 8 (44.4) | 54 (33.3) |
|  | 5.6.1 Currently using - Specified purpose | 2 (11.1) | 31 (19.1) |
|  | 5.6.2 Currently using - Unspecified purpose | - | 5 (3.1) |
|  | 5.6.3 Currently using - With another substance/prescription | 1 (5.6) | 7 (4.3) |
|  | 5.6.4 Currently using - Side effects | - | 3 (1.9) |
|  | 5.6.5 Currently using - Desires to stop use | - | 1 (0.6) |
|  | 5.6.6 Currently using - Agrees to stop or reduce use | - | 2 (1.2) |
|  | 5.6.7 Currently using - Perceived negative outcome from non-use | - | 1 (0.6) |
|  | 5.6.8 Currently using - Ineffective or inconsistent | - | 6 (3.7) |
|  | 5.6.9 Not currently using - Ineffective or inconsistent | 1 (5.6) | 4 (2.5) |
|  | 5.6.10 Not currently using - Perceived negative outcome from use | 1 (5.6) | 3 (1.9) |
|  | 5.6.10 Not currently using - Medication/treatment access | 2 (11.1) | 2 (1.2) |
|  | 5.6.11 Not currently using - Employment | - | 1 (0.6) |
|  | 5.6.12 Not currently using - Unspecified purpose | 1 (5.6) | 2 (1.2) |
|  | 5.6.13 Not currently using - Current condition | - | 1 (0.6 |
|  | 5.7 Complaining about marijuana | - | 6 (3.7) |
|  | 5.7.1 Complaint - Not related to own use | - | 2 (1.2) |
|  | 5.7.2 Complaint - Medical marijuana access process | - | 4 (2.5) |
|  | 5.8 Not enough context to determine purpose | - | 1 (0.6) |
|  | 5.9 Cost Prohibitive | 2 (11.1) | 6 (3.7) |
|  | 5.10 Request to change marijuana mention in EHR notes | - | 3 (1.9) |
|  | 5.11 Resources for MMJ | - | 6 (3.7) |
|  | 5.11.1 Research/Studies | - | 4 (2.5) |
|  | 5.11.2 Personal anecdote | - | 2 (1.2) |
|  | 5.12 Patient-related MMJ documentation | - | 7 (4.3) |
|  | 5.12.1 Perceived need for integration of care | - | 4 (2.5) |
|  | 5.12.2 Proof of qualification | - | 3 (1.9) |
|  | 5.13 Acknowledgement of provider perspective | - | 4 (2.5) |
|  | 5.14 Case management for medical marijuana | - | 1 (0.6) |
|  | 5.15 Statement of plan to use | - | 17 (10.5) |
|  | 5.15.1 Statement of plan - Use with pain medication | - | 2 (1.2) |
|  | 5.15.2 Statement of plan - Substitute for pain medication | - | 5 (3.1) |
|  | 5.15.3 Statement of plan - Approved to use | - | 4 (2.5) |
|  | 5.15.4 Statement of plan - Ordered product | - | 2 (1.2) |
|  | 5.15.5 Statement of plan - Certification | - | 2 (1.2) |
|  | 5.15.6 Statement of plan - Substitute for anxiety medication | - | 3 (1.9) |
|  | 5.16 Debating use | - | 4 (2.5) |
|  | 5.17 Statement of preference over medications | - | 2 (1.2) |

Provider code frequencies

|  | Code Category | 2012-2016 (n=20), n (%) | 2017-2022 (n=80), n (%) |
| --- | --- | --- | --- |
|  |  |  |  |
| **1 Correctly classified message** | |  |  |
|  | 1.1 Yes | 17 (85.0) | 80 (100.0) |
|  | 1.2 No, not related to marijuana | 3 (15.0) | - |
|  | 1.3 No, reference to Rx | - | - |
| **2 Message recipient** | |  |  |
|  | 2.1 Patient | 17 (100.0) | 75 (93.8) |
|  | 2.2 Non-patient | - | 5 (6.3) |
| **3 Reason for use** | |  |  |
|  | 3.1 Unspecified | 14 (82.4) | 65 (81.3) |
|  | 3.2 Pain | 2 (11.8) | 8 (12.3) |
|  | 3.2.1 Pain (unspecified) | 1 (5.9) | 7 (8.8) |
|  | 3.2.2 Neck Pain | 1 (5.9) | - |
|  | 3.2.3 Chronic Refractory Pain | - | 1 (1.3) |
|  | 3.3 Sleep | - | 1 (1.3) |
|  | 3.4 Anxiety | - | 4 (5.0) |
|  | 3.5 Inflamed skin issues | - | 1 (1.3) |
|  | 3.6 Seizure | - | 1 (1.3) |
|  | 3.7 Inflammation | - | 1 (1.3) |
|  | 3.8 Headaches | - | 1 (1.3) |
|  | 3.9 Glioblastoma | 1 (5.9) | - |
| **4 Purpose of cannabis mention** | |  |  |
|  | 4.1 Provide guidance on patient use | 3 (17.6) | 56 (70.0) |
|  | 4.1.1 Recommendation - Use marijuana | - | 4 (5.0) |
|  | 4.1.2 Recommendation - Stop using marijuana | - | 3 (3.8) |
|  | 4.1.3 Explanation - Positive effects of use | - | 3 (3.8) |
|  | 4.1.4 Assistance - Medical marijuana access | - | 22 (27.5) |
|  | 4.1.5 Explanation - Drug Interaction | 2 (11.8) | 12 (15.0) |
|  | 4.1.6 Explanation - Negative effects of use | 1 (5.9) | 8 (10.0) |
|  | 4.1.7 Explanation - Insurance coverage | 1 (5.9) | 6 (7.5) |
|  | 4.1.8 Recommendation - Do not use | - | 4 (5.0) |
|  | 4.1.9 Recommendation - Time of use | - | 4 (5.0) |
|  | 4.1.10 Explanation - No effect of use | - | 1 (1.3) |
|  | 4.2 Unable to refer, prescribe, or recommend use | 2 (11.8) | 21 (28.3) |
|  | 4.2.1 Legal | 2 (11.8) | - |
|  | 4.2.2 Geisinger policy | - | 8 (10.0) |
|  | 4.2.3 Outside scope | - | 7 (8.8) |
|  | 4.2.4 Unspecified | - | 4 (5.0) |
|  | 4.2.5 Lack of evidence for efficacy | - | 2 (2.5) |
|  | 4.2.6 Unavailability | - | 1 (1.3) |
|  | 4.3 Provide information on marijuana/cannabis screening | 13 (76.5) | 7 (8.8) |
|  | 4.3.1 Inform - Screening ordered | 1 (5.9) | - |
|  | 4.3.2 Inform - Positive result | 11 (64.7) | 7 (8.8) |
|  | 4.3.3 Inform - Medication use agreement violation | 7 (41.2) | 4 (5.0) |
|  | 4.3.4 Inform - Educating about screening | - | 1 (1.3) |
|  | 4.4 Inquiry about patient use | 2 (11.8) | 10 (12.5) |
|  | 4.4.1 Inquiry - Use or exposure | 1 (5.9) | 2 (2.5) |
|  | 4.4.2 Inquiry - Stopped using | 1 (5.9) | 1 (1.3) |
|  | 4.4.3 Inquiry - Effects of use | - | 3 (3.8) |
|  | 4.4.4 Inquiry - Appointment | - | 1 (1.3) |
|  | 4.4.5 Inquiry - Reason for use | - | 2 (2.5) |
|  | 4.4.6 Inquiry - Dosage | - | 2 (2.5) |
|  | 4.5 Provider-to-Provider Consideration | 1 (5.9) | 3 (3.8) |
|  | 4.6 Mention of current use status | 1 (5.9) | 3 (3.8) |
|  | 4.7 Statement of uncertainty | - | 5 (6.3) |
|  | 4.8 Research on medical marijuana | 1 (5.9) | 3 (3.8) |
|  | 4.8.1 Inform on current state of research | - | 2 (2.5) |
|  | 4.8.2 Suggestion to self-educate | - | 1 (1.3) |
|  | 4.8.3 Plan to self-education | 1 (5.9) | - |
|  | 4.9 Request to see medical marijuana card | - | 2 (2.5) |
