# Supplementary material for: Discussions of Cannabis Over Patient Portal Secure Messaging: Content Analysis
Source: J Med Internet Res. 2024 Dec 12;26:e63311. doi: 10.2196/63311 (PMC11671783; doi:10.2196/63311)
Supplement: Multimedia Appendix 7 [file jmir_v26i1e63311_app7.docx]

Access to medical marijuana in Pennsylvania requires a patient to follow several steps:

1) The patient must receive a medical marijuana certification from a provider who has been licensed as a medical marijuana certifier. A medical marijuana certification requires that the patient is at least 18 years old and has at least 1 of 24 qualifying medical conditions. The certification validates the patient’s basic information and that they are eligible for the state’s medical marijuana program based on an evaluation by the certifying provider. The certification includes the patient’s basic personal information, the qualifying conditions that make the patient eligible, the issuance and expiration date of the certification, and the information of the certifying provider.

2) Once a patient acquires a medical marijuana certification, they can apply for a medical marijuana card. Medical marijuana cards are administered by the state of Pennsylvania, cost $50, and can be renewed annually so long as the patient remains eligible for the program.

3) Once a patient has acquired a medical marijuana card, they can purchase medical marijuana products from licensed dispensaries in Pennsylvania.

**Reference**

How to get a Medical Marijuana Card in Pennsylvania. Pennsylvania Cannabis Information. Updated January 2024. Accessed March 25, 2024. https://pennsylvaniastatecannabis.org/mmj-card
