# Supplementary material for: Discussions of Cannabis Over Patient Portal Secure Messaging: Content Analysis
Source: J Med Internet Res. 2024 Dec 12;26:e63311. doi: 10.2196/63311 (PMC11671783; doi:10.2196/63311)
Supplement: Multimedia Appendix 8 [file jmir_v26i1e63311_app8.docx]

| 2016 | 2017 | 2018 | 2019 | 2020 | 2021 | 2022 | 2023 | 2024 |
| --- | --- | --- | --- | --- | --- | --- | --- | --- |
| Amyotrophic Lateral Sclerosis | Amyotrophic Lateral Sclerosis | Amyotrophic Lateral Sclerosis | Amyotrophic Lateral Sclerosis | Amyotrophic Lateral Sclerosis | Amyotrophic Lateral Sclerosis | Amyotrophic Lateral Sclerosis | Amyotrophic Lateral Sclerosis | Amyotrophic Lateral Sclerosis |
|  |  |  | Anxiety disorders | Anxiety disorders | Anxiety disorders | Anxiety disorders | Anxiety disorders | Anxiety disorders |
| Autism | Autism | Autism | Autism | Autism | Autism | Autism | Autism | Autism |
| Cancer, including remission therapy | Cancer, including remission therapy | Cancer, including remission therapy | Cancer, including remission therapy | Cancer, including remission therapy | Cancer, including remission therapy | Cancer, including remission therapy | Cancer, including remission therapy | Cancer, including remission therapy |
|  |  |  |  |  |  |  | Chronic Hepatitis C | Chronic Hepatitis C |
| Crohn’s Disease | Crohn’s Disease | Crohn’s Disease | Crohn’s Disease | Crohn’s Disease | Crohn’s Disease | Crohn’s Disease | Crohn’s Disease | Crohn’s Disease |
| Damage to the nervous tissue of the central nervous system with objective neurological indication of intractable spasticity, and other associated neuropathies | Damage to the nervous tissue of the central nervous system with objective neurological indication of intractable spasticity, and other associated neuropathies | Damage to the nervous tissue of the central nervous system with objective neurological indication of intractable spasticity, and other associated neuropathies | Damage to the nervous tissue of the central nervous system with objective neurological indication of intractable spasticity, and other associated neuropathies | Damage to the nervous tissue of the central nervous system with objective neurological indication of intractable spasticity, and other associated neuropathies | Damage to the nervous tissue of the central nervous system with objective neurological indication of intractable spasticity, and other associated neuropathies | Damage to the nervous tissue of the central nervous system with objective neurological indication of intractable spasticity, and other associated neuropathies | Damage to the nervous tissue of the central nervous system with objective neurological indication of intractable spasticity, and other associated neuropathies | Damage to the nervous tissue of the central nervous system with objective neurological indication of intractable spasticity, and other associated neuropathies |
|  |  | Dyskinetic and spastic movement disorders | Dyskinetic and spastic movement disorders | Dyskinetic and spastic movement disorders | Dyskinetic and spastic movement disorders | Dyskinetic and spastic movement disorders | Dyskinetic and spastic movement disorders | Dyskinetic and spastic movement disorders |
| Epilepsy | Epilepsy | Epilepsy | Epilepsy | Epilepsy | Epilepsy | Epilepsy | Epilepsy | Epilepsy |
| Glaucoma | Glaucoma | Glaucoma | Glaucoma | Glaucoma | Glaucoma | Glaucoma | Glaucoma | Glaucoma |
| HIV/AIDS | HIV/AIDS | HIV/AIDS | HIV/AIDS | HIV/AIDS | HIV/AIDS | HIV/AIDS | HIV/AIDS | HIV/AIDS |
| Huntington’s Disease | Huntington’s Disease | Huntington’s Disease | Huntington’s Disease | Huntington’s Disease | Huntington’s Disease | Huntington’s Disease | Huntington’s Disease | Huntington’s Disease |
| Inflammatory Bowel Disease | Inflammatory Bowel Disease | Inflammatory Bowel Disease | Inflammatory Bowel Disease | Inflammatory Bowel Disease | Inflammatory Bowel Disease | Inflammatory Bowel Disease | Inflammatory Bowel Disease | Inflammatory Bowel Disease |
| Intractable Seizures | Intractable Seizures | Intractable Seizures | Intractable Seizures | Intractable Seizures | Intractable Seizures | Intractable Seizures | Intractable Seizures | Intractable Seizures |
| Multiple Sclerosis | Multiple Sclerosis | Multiple Sclerosis | Multiple Sclerosis | Multiple Sclerosis | Multiple Sclerosis | Multiple Sclerosis | Multiple Sclerosis | Multiple Sclerosis |
|  |  | Neurodegenerative diseases | Neurodegenerative diseases | Neurodegenerative diseases | Neurodegenerative diseases | Neurodegenerative diseases | Neurodegenerative diseases | Neurodegenerative diseases |
| Neuropathies | Neuropathies | Neuropathies | Neuropathies | Neuropathies | Neuropathies | Neuropathies | Neuropathies | Neuropathies |
|  |  | Opioid use disorder for which conventional therapeutic interventions are contraindicated or ineffective, or for which adjunctive therapy is indicated in combination with primary therapeutic interventions | Opioid use disorder for which conventional therapeutic interventions are contraindicated or ineffective, or for which adjunctive therapy is indicated in combination with primary therapeutic interventions | Opioid use disorder for which conventional therapeutic interventions are contraindicated or ineffective, or for which adjunctive therapy is indicated in combination with primary therapeutic interventions | Opioid use disorder for which conventional therapeutic interventions are contraindicated or ineffective, or for which adjunctive therapy is indicated in combination with primary therapeutic interventions | Opioid use disorder for which conventional therapeutic interventions are contraindicated or ineffective, or for which adjunctive therapy is indicated in combination with primary therapeutic interventions | Opioid use disorder for which conventional therapeutic interventions are contraindicated or ineffective, or for which adjunctive therapy is indicated in combination with primary therapeutic interventions | Opioid use disorder for which conventional therapeutic interventions are contraindicated or ineffective, or for which adjunctive therapy is indicated in combination with primary therapeutic interventions |
| Parkinson’s Disease | Parkinson’s Disease | Parkinson’s Disease | Parkinson’s Disease | Parkinson’s Disease | Parkinson’s Disease | Parkinson’s Disease | Parkinson’s Disease | Parkinson’s Disease |
| Post-traumatic stress disorder | Post-traumatic stress disorder | Post-traumatic stress disorder | Post-traumatic stress disorder | Post-traumatic stress disorder | Post-traumatic stress disorder | Post-traumatic stress disorder | Post-traumatic stress disorder | Post-traumatic stress disorder |
| Severe chronic or intractable pain | Severe chronic or intractable pain | Severe chronic or intractable pain | Severe chronic or intractable pain | Severe chronic or intractable pain | Severe chronic or intractable pain | Severe chronic or intractable pain | Severe chronic or intractable pain | Severe chronic or intractable pain |
| Sickle Cell Anemia | Sickle Cell Anemia | Sickle Cell Anemia | Sickle Cell Anemia | Sickle Cell Anemia | Sickle Cell Anemia | Sickle Cell Anemia | Sickle Cell Anemia | Sickle Cell Anemia |
|  |  | Terminal illness | Terminal illness | Terminal illness | Terminal illness | Terminal illness | Terminal illness | Terminal illness |
|  |  |  | Tourette syndrome | Tourette syndrome | Tourette syndrome | Tourette syndrome | Tourette syndrome | Tourette syndrome |
